# Emodin Protects SH-SY5Y Cells Against Zinc-Induced Synaptic Impairment and Oxidative Stress Through the ERK1/2 Pathway

**DOI:** 10.3389/fphar.2022.821521

**Published:** 2022-02-07

**Authors:** Qian Chen, Chencen Lai, Fa Chen, Yuanting Ding, Yiyuan Zhou, Songbai Su, Ruiqing Ni, Zhi Tang

**Affiliations:** ^1^ Department of Obstetrics, The First Affiliated Hospital of Guizhou University of Traditional Chinese Medicine, Guiyang, China; ^2^ Clinical Research Center, The First Affiliated Hospital of Guizhou University of Traditional Chinese Medicine, Guiyang, China; ^3^ Preparation Center, The First Affiliated Hospital of Guizhou University of Traditional Chinese Medicine, Guiyang, China; ^4^ Department of Miao Medicine, The First Affiliated Hospital of Guizhou University of Traditional Chinese Medicine, Guiyang, China; ^5^ Institute for Regenerative Medicine, University of Zurich, Zurich, Switzerland; ^6^ Institute for Biomedical Engineering, ETH Zurich and University of Zurich, Zurich, Switzerland; ^7^ Key Laboratory of Endemic and Ethnic Diseases, Ministry of Education and Key Laboratory of Medical Molecular Biology of Guizhou Province, Guizhou Medical University, Guiyang, China

**Keywords:** emodin, ERK1/2 pathway, mitochondria, oxidative stress, SH-SY5Y cells, synaptic impairment, zinc

## Abstract

Zinc is an essential trace element important for the physiological function of the central nervous system. The abnormal accumulation of zinc inside neurons may induce mitochondrial dysfunction and oxidative stress, which contribute to many brain diseases. We hypothesized that natural anthraquinone derivative emodin can protect against neurotoxicity induced by pathological concentrations of zinc via the extracellular signal-regulated kinase 1/2 (ERK1/2) signaling pathway and alleviate oxidative stress and mitochondrial dysfunction. Human neuroblastoma (SH-SY5Y 26 cells) was treated with zinc sulfate and different concentrations of emodin, and changes in the levels of ETK1/2 expression, oxidative stress (DCFH-DA staining), mitochondrial function (JC-1 staining), lipid peroxidation (4-hydroxynonenal staining), and DNA oxidation (8-hydroxy-2-deoxyguanosine staining) were examined. Emodin ameliorated zinc-induced altered expression of levels of phosphorylated ERK1/2 (not total ETK1/2) and synaptic proteins (presynaptic SNAP 25, synaptophysin and postsynaptic PSD95) in SH-SY5Y cells. Moreover, emodin inhibited the generation of reactive oxygen species and oxidative stress and facilitated the collapse of mitochondrial membrane potential (ΔΨm) in SH-SY5Y cells. In conclusion, our results indicated that emodin exerts neuroprotective effects against zinc by normalizing synaptic impairment by decreasing the phosphorylation of ERK1/2, reducing reactive oxygen species and protecting mitochondrial function.

## Introduction

Zinc is an essential trace element obtained from the diet that regulates the expression of many biological molecules and the activation of signaling pathways. Zinc deficiency affects up to 2 billion people worldwide and has profound effects on immune and neurological system functions (Kambe et al., 2015). In the central nervous system, zinc is one of the most abundant oligoelements and is involved in the balance of excitatory and inhibitory signals of synapses (Frederickson et al., 2005; Sensi et al., 2009; Sensi et al., 2018). During neuronal activity, zinc is released in the form of free ions (Zn^2+^) from synaptic vesicles. Maintaining the homeostasis of zinc is thus essential for the physiological function of the brain (Frederickson et al., 2005; Mocchegiani et al., 2005). Excessive zinc in the extracellular fluid has been shown to increase neurotoxicity and induce mitochondrial dysfunction and oxidative stress (Greenough et al., 2013; Wang et al., 2020). Abnormal increases in the levels of metal ions, including Zn^2+^, have been found in Aβ and form Aβ–Zn complexes (Miller et al., 2010), which results in a loss of zinc modulatory activity and cognitive deficits in animal models of Alzheimer’s disease (AD) (Deshpande et al., 2009).

In addition, zinc accumulation has been shown to cause mitochondrial dysfunction and oxidative stress in AD (Sensi et al., 2003; Liu et al., 2021) as well as in ischemic stroke models (Ji et al., 2018; Zhao et al., 2018). Mitochondrial Zn^2+^ accumulation is a possible trigger of hippocampal ischemic injury (Ji et al., 2018). The synergistic interaction between Zn^2+^ and reactive oxygen species (ROS) has been shown to amplify ischemic brain injury in rodent models (Zhao et al., 2018) through direct ROS generation or through mitochondrial Ca^2+^ uniporters (Pivovarova et al., 2014). A recent study indicated that zinc status is introduced through inflammation through the NOD-, LRR- and pyrin domain-containing protein 3 (NLRP3)-mediated pathway (Rivers-Auty et al., 2021).

Emodin, an anthraquinone derivative, is a major active ingredient of many herbs, including Rheum palmatum, Polygonum cuspidatum, Aloe vera, and Cassia obtusifolia (Dong et al., 2016). Emodin shows neuroprotective and anti-inflammatory effects in animal models of cerebral ischemia stroke, traumatic brain injury, AD, and Parkinson’s disease (Li et al., 2014; Ahn et al., 2016; Fan et al., 2018; Chao et al., 2020). Different signaling pathways have been reported to mediate the effect of emodin, such as the nuclear factor-erythroid factor 2-related factor 2 (Nrf2), phosphatidylinositol 3-kinase/Beclin-1/B-cell lymphoma 2, and adenosine monophosphate (AMP)-activated protein kinase signaling pathways (Du et al., 2019; Liu et al., 2019; Li et al., 2021). Previous studies from our and other groups have reported that emodin demonstrates a neuroprotective effect (Gu et al., 2005; Liu et al., 2010a; Liu et al., 2010b; Park et al., 2016; Fan et al., 2018), and it can inhibit the neurotoxic effect of NaF on SH-SY5Y cells by reducing ROS overproduction and oxidative stress (Lai et al., 2020). The underlying mechanism of emodin remains to be elucidated.

Extracellular signal-regulated kinase 1/2 (ERK1/2) is activated by neurotrophins and other chemicals and plays an important role in the differentiation, survival, structural plasticity, and long-term potentiation of neurons, as well as memory formation in animal models (Schafe et al., 2000). Emerging evidence suggests that the ERK1/2 signaling pathway is implicated in a number of neurodegenerative diseases with oxidative stress (Perry et al., 1999). Aberrant accumulation of activated ERK1/2 in neurons has been reported in AD brains (Pei et al., 2002; Pei et al., 2003). Here, we hypothesized that emodin can protect against neurotoxicity induced by pathological concentrations of zinc via the ERK1/2 signaling pathway and alleviate oxidative stress and mitochondrial dysfunction. We exposed human neuroblastoma SH-SY5Y cells to a high dose of zinc sulfate and assessed the effect of emodin on attenuating synaptic impairment, mitochondrial function and oxidative stress damage.

## Materials and Methods

### Materials and Antibodies

Emodin with purity >96% was purchased from the National Institutes for Food and Drug Control (China). Zinc sulfate was purchased from Sigma-Aldrich (United States). Total ERK (1:1,000) and phosphorylated ERK (1:1,000) antibodies were purchased from Cell Signaling Technology (Boston, Massachusetts). Antibodies against PSD95 (1:1,000), SNAP25 (1:1,000), synaptophysin (1:1,000), 4-hydroxynonenal (4-HNE, 1:100), and 8-hydroxy-2′-deoxyguanosine (8-OHdG, 1:200) were purchased from Abcam (United States). Anti-mouse and anti-rabbit secondary antibodies (1:5,000) were purchased from Bio–Rad (United States). Anti-rabbit DyLight-546 and anti-mouse DyLight 488 secondary antibodies were purchased from Invitrogen (California, United States). A cell counting kit-8 for detecting the levels of cell death, ROS assay kit with 2′-7′dichlorofluorescin diacetate (DCFH-DA) for detecting intracellular hydrogen peroxide (H_2_O_2_), and oxidative stress and a mitochondrial membrane potential assay kit with JC-1 were purchased from Beyotime (China).

### Cell Culture and Treatment

SH-SY5Y cells were cultured in Dulbecco’s modified Eagle’s dedium (DMEM)/nutrient mixture F-12 (F12) supplemented with 10% fetal bovine serum (FBS) at 37°C. At 80% confluence, cells were seeded into 6-well culture plates. After serum deprivation overnight, different concentrations of emodin (10, 25, and 40 μM) were applied to pretreat the cells for 2 h in serum-free media. Then, 300 μM zinc sulfate was applied for an additional 4 h.

### Neurotoxicity Experiments

The level of cell death was analysed using a cell counting kit-8. In brief, SH-SY5Y cells were seeded into 96-well plates at nearly 5 × 10^4^ cells per well in 100 μl culture medium. Cells were treated with various concentrations of emodin (0, 10, 25 and 40 μM) for 6 h and various concentrations of emodin (0, 10, 25 and 40 μM) for 2 h prior to the addition of ZnSO_4_ (300 μM) for 4 h. Ten microliters of CCK-8 was added to each well. After 1 h of incubation, the absorbances (450 nm) of the treated samples were measured against a blank control in a Thermo Scientific Multiskan FC Microplate Reader (Thermo Fisher Scientific).

### Cell Extraction and Western Blotting Analysis

After being washed with cold phosphate-buffered saline (PBS, pH 7.4), the cells were lysed with 100 μL preheated sodium dodecyl sulfate (SDS) sample buffer and scraped with a rubber policeman. The extract was placed in an Eppendorf tube, boiled for 5 min, and cooled on ice immediately. Cell lysates (10 μl) were run on TGX Stain-Free-FastCast Acrylamide gels (Bio–Rad). The gels were stain-free activated for 45 s and imaged utilizing the ChemiDoc MP imaging system (Bio–Rad, United States). The separated proteins were transferred to polyvinylidene difluoride (PVDF) membranes utilizing the Trans-Blot Turbo Transfer System (Bio–Rad), and the membranes were blocked with 5% nonfat dried milk in Tris-buffered saline (TBS) for 1 h. The blocking solution was then removed from the incubation solution, and a specific antibody was added and incubated overnight at 4°C. Immunoblot images were obtained by a ChemiDoc MP imaging system. The stain-free signals and immunoblots were evaluated using ImageLab software (Bio–Rad). Protein expression levels were normalized to the stain-free total protein lane density obtained from each gel.

### ROS Measurements

To detect intracellular H_2_O_2_ and oxidative stress, DCFH-DA staining was used. Cells in 24-well plates at a density of 5 × 10^4^/well were incubated in control media or 300 μM zinc sulfate for 4 h with or without 2 h of emodin pretreatment (10, 25, and 40 μM). DCFH-DA staining was used to determine changes in intracellular ROS levels according to the manufacturers’ instructions. Briefly, the cells were maintained in DCFH-DA at 37°C for 20 min, the staining solution was removed, and the cells were gently washed twice with PBS pH 7.4. Image analysis was performed using a confocal microscope (Leica SP8, Leica, Germany) and ImageJ 1.49 (NIH, United States).

### Mitochondrial Membrane Potential Detection

Mitochondrial membrane potential was measured using the mitochondrial membrane potential probe JC-1 staining dye in SH-SY5Y cells. Briefly, cells in 24-well plates were treated with 300 μM zinc sulfate for 4 h with or without pretreatment with emodin (10, 25, and 40 μM) for 2 h. After adding F12 medium/JC-1 working solution (1:1), the cells were maintained in a CO2 incubator for 20 min. The staining solution was removed, and then the cells were gently washed twice with JC-1 staining buffer. Fluorescence was detected with confocal microscopy (Leica SP8). The pictures were captured in five fields of each sample in triplicate. The ΔΨm of SH-SY5Y cells was represented by the ratio of monomeric JC-1 to aggregated JC-1.

### Immunofluorescence Staining

SH-SY5Y cells grown on coverslips were treated with 300 μM zinc sulfate for 4 h with or without pretreatment with emodin (10, 25, and 40 μM) for 2 h. The cells were washed with PBS pH 7.4 and fixed with 4% paraformaldehyde for 20 min. After permeation with TBS containing 0.1% Triton X-100 for 5 min, the cells were blocked with 5% bovine serum albumin (BSA) in TBS for 30 min. The cells were then incubated with primary antibodies against 4-HNE (1:100) or 8-OHdG (1:200) overnight at 4°C, and anti-rabbit DyLight-546 and anti-mouse DyLight-488 secondary antibodies (1:200) were incubated for 1 h in the dark. Nuclei were counterstained with 4′,6-diamidino-2-phenylindole (DAPI). Image analysis was performed using a confocal microscope (Leica SP8) and ImageJ 1.49V (NIH, United States).

### Statistical Analysis

Welch’s *t* test was used for two-group comparisons in the CCK-8 assay. One-way ANOVA and Bonferroni post hoc analysis were used for multiple-group comparisons using GraphPad Prism 8 (GraphPad Prism). Data are presented as the mean ± standard error (SEM) (n = 3). Significance was set at *p* < 0.05.

## Results

### Emodin Protected SH-SY5Y Cells Against Zinc-Induced Cell Death

**FIGURE 1:**
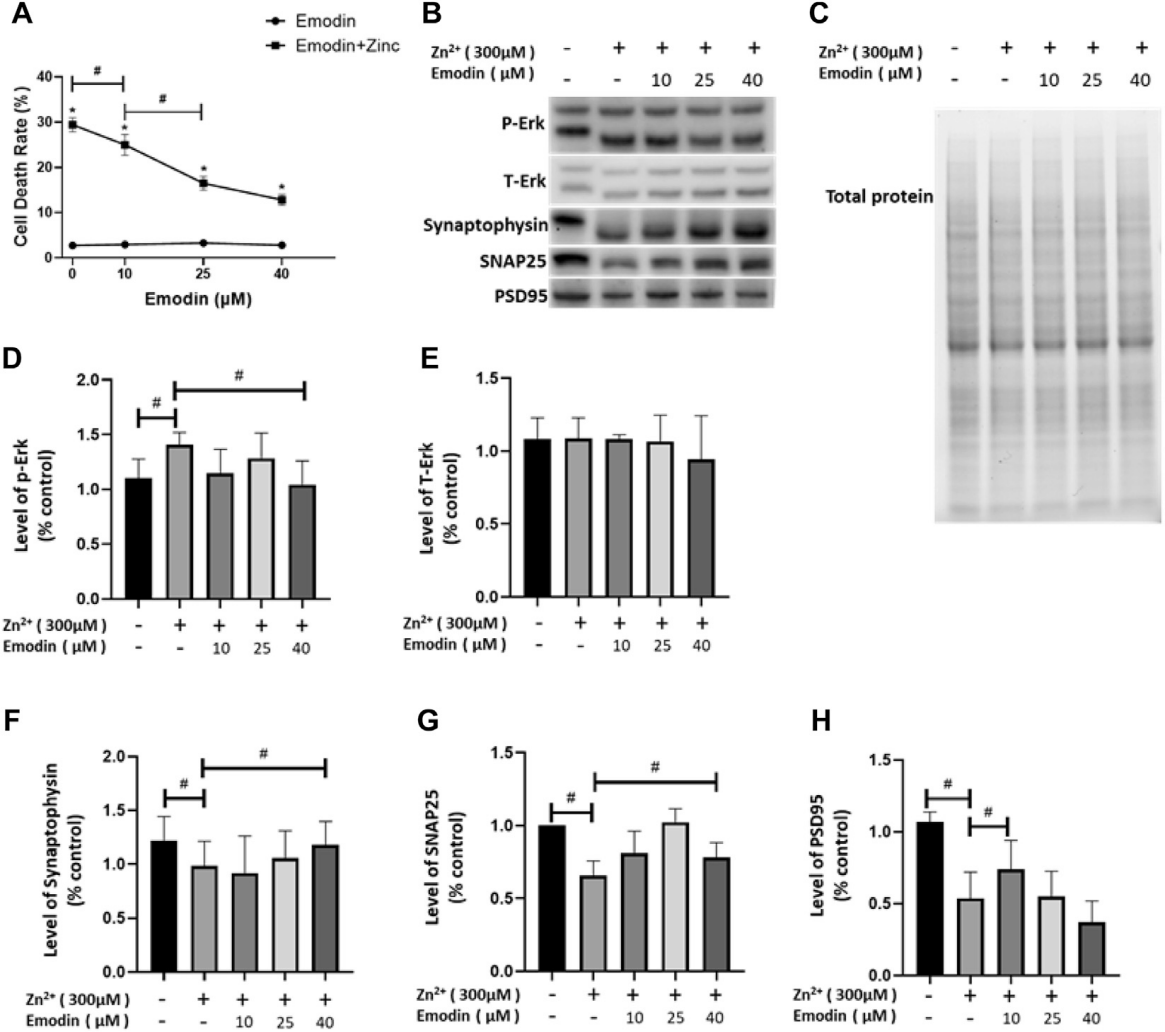
Emodin attenuates cell death, reverses the activation of phosphorylated ERK1/2 and affects synapse-related proteins in SH-SY5Y cells exposed to 300 μM zinc sulfate. **(A)** The cell death rate determined using the CCK-8 kit. **(B)** Immunoblot image of phosphorylated ERK1/2 (p-Erk) and total ERK1/2 (t-ERK), synaptophysin, SNAP25, and PSD95 in SH-SY5Y cells. **(C)** Stain-free image of SH-SY5Y cell lysate. **(D, E)** Immunoblot analysis of phosphorylated ERK1/2 and total ERK1/2. **(F–H)** Immunoblot analysis of synapse-related proteins. Different concentrations of emodin (10, 25, and 40 μM) were applied. One-way ANOVA and Bonferroni post hoc analysis, #*p* < 0.05; Welch’s *t* test, *p < 0.05 vs. SH-SY5Y cells treated with emodin only; ns, not significant. Data are presented as the mean ± SEM.

To investigate whether emodin is effective against neurotoxicity, the cell death of SH-SY5Y cells treated with different concentrations of emodin for 6 h and with various concentrations of emodin for 2 h, followed by exposure to ZnSO_4_ for 4 h, was examined. Compared with the control cells, the cell death rate of SH-SY5Y cells treated with emodin for only 6 h showed no significant differences (Figure 1A). Exposure to 300 μM ZnSO_4_ for 4 h resulted in a vital increase in SH-SY5Y cell death, while pretreatment with emodin prior to the addition of ZnSO_4_ significantly decreased the level of SH-SY5Y cell death. Therefore, emodin might play a protective role in SH-SY5Y cells against zinc-induced cell death.

### Emodin Attenuated the Phosphorylation of ERK1/2 in SH-SY5Y Cells

We hypothesized that emodin protects SH-SY5Y cells against zinc via the ERK1/2 pathway. We examined the phosphorylated and total expression levels of ERK in SH-SY5Y cell lysates by using western blotting. Zinc sulfate (300 μM) significantly increased the expression levels of phosphorylated ERK1/2 (*p* = 0.03) but not total ERK1/2 in the treated SH-SY5Y cells compared to the control group (Figures 1B–E). Pretreatment with emodin at 40 μM (but not at 10 or 25 μM) completely abolished the zinc sulfate-induced increase in the levels of phosphorylated ERK1/2 (*p* = 0.02, vs. Zn-treated group) (Figures 1B,D). No difference was observed in the level of total ERK1/2 after pretreatment with emodin at different doses.

### Emodin Ameliorated Synaptic Impairment in Zinc Sulfate-Treated SH-SY5Y Cells

To investigate the possible effects of emodin on synaptic function-related proteins affected by the presence of zinc, we examined the changes in the levels of presynaptic terminal proteins (SNAP25 and synaptophysin) and postsynaptic density protein (PSD95). We found that 300 μM zinc sulfate treatment significantly reduced the expression levels of presynaptic SNAP25 (*p* = 0.04), synaptophysin (*p* = 0.01) and postsynaptic PSD95 (approximately 50%, *p* = 0.04) in SH-SY5Y cells compared to the control group (Figures 1F,G,H). Pretreatment with emodin at 40 μM (but not at 10 or 25 μM) significantly ameliorated the reduction in the levels of synaptophysin (*p* = 0.04) and SNAP25 (*p* = 0.03) induced by zinc sulfate compared to the zinc-treated control group (Figures 1F,G). Pretreatment with emodin at 10 μM (but not at 25 or 40 μM) significantly ameliorated the zinc-induced reduction in the levels of PSD95 by approximately 50% (*p* = 0.01) compared to the zinc-treated control group (Figure 1H).

### Emodin Inhibited ROS Generation in Zinc-Treated SH-SY5Y Cells

**FIGURE 2:**
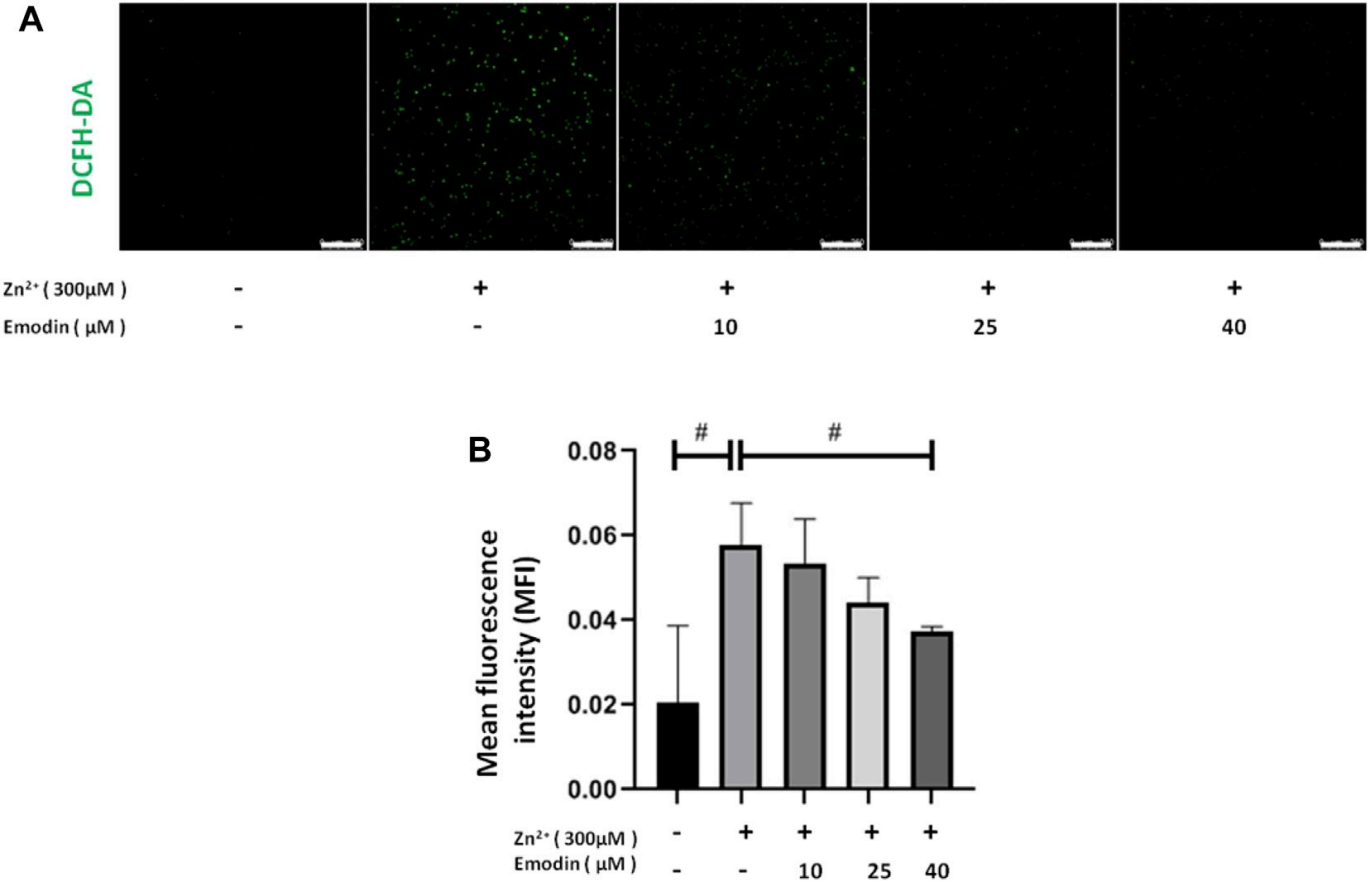
Emodin decreased zinc-induced reactive oxygen species production in SH-SY5Y cells. **(A)** Fluorescence staining of DCFH-DA in SH-SY5Y cells. From left to right are untreated cells and 300 μM zinc sulfate-treated cells without emodin pretreatment (10, 25, and 40 μM). Scale bar = 250 μm. **(B)** Mean fluorescence intensity analysis. One-way ANOVA and Bonferroni post hoc analysis, #*p* < 0.05; Data are presented as the mean ± SEM.

To detect zinc-induced oxidative stress and the antioxidant effect of emodin, we assessed ROS production by using DCFH-DA fluorescent staining in SH-SY5Y cells. Treatment with 300 μM zinc sulfate significantly increased the intracellular level of ROS, as indicated by the DCFH-DA fluorescence intensity, in SH-SY5Y cells compared to the control group (*p* = 0.009). Pretreatment with emodin at 40 μM significantly decreased the zinc-induced increase in DCFH-DA fluorescence intensity by approximately 50% (*p* = 0.04 vs. zinc-treated control group) (Figures 2A,B).

### Emodin Reestablished the Loss of Mitochondrial Membrane Potential (ΔΨm) in Zinc-Treated SH-SY5Y Cells

**FIGURE 3:**
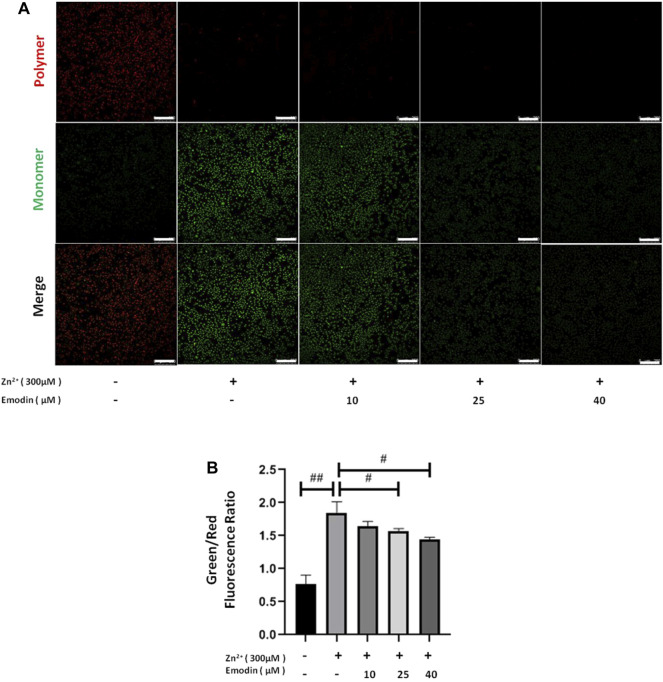
Emodin corrected the effects of zinc on the ΔΨm depolarization in SH-SY5Y cells. **(A)** Fluorescence staining of JC-1 in untreated and zinc-treated SH-SY5Y cells with or without pretreatment with emodin (10, 25, and 40 μM). Mitochondrial aggregate, polymer form of JC-1 (red) indicating normal ΔΨm, and monomeric form of JC-1 (green) indicating dissipation of ΔΨm. Scale bar = 250 μm. **(B)** The ratio of green fluorescence to red fluorescence. One-way ANOVA and Bonferroni post hoc analysis; #*p* < 0.05, ##*p* < 0.001 significant. Data are presented as the mean ± SEM.

Next, we examined whether emodin can ameliorate the mitochondrial dysfunction induced by zinc treatment. We measured the ΔΨm in zinc-treated SH-SY5Y cells by using JC-1 staining. JC-1 aggregates in the normal mitochondrial matrix emit red fluorescence. Green fluorescence is produced in JC-1-stained cells following the loss of ΔΨm. The ratio of green and red fluorescence was used to indicate the toxicity introduced by zinc. in mitochondria and the protective effect of emodin. In the control groups, JC-1 aggregated in mitochondria, and the green/red fluorescence intensity ratio was 0.77 ± 0.11. Exposure of SH-SY5Y cells to 300 μM zinc sulfate for 4 h increased the green/red fluorescence intensity ratio to 1.85 ± 0.14 (*p* = 0.0008, vs. control), implying the collapse of ΔΨm (Figure 3). In the presence of increasing concentrations of emodin, the green fluorescence gradually weakened, while the red fluorescence remained the same, resulting in a reduced green/red fluorescence intensity ratio. The green/red fluorescence intensity ratio from JC-1 staining was 1.57 ± 0.03 at 25 μM (*p* = 0.04, vs. zinc-treated control group) and 1.44 ± 0.03 at 40 μM (*p* = 0.004, vs. zinc-treated control group), implying the reestablishment of ΔΨm.

### Emodin Attenuated Oxidative Stress Damage in Zinc-Treated SH-SY5Y Cells

**FIGURE 4:**
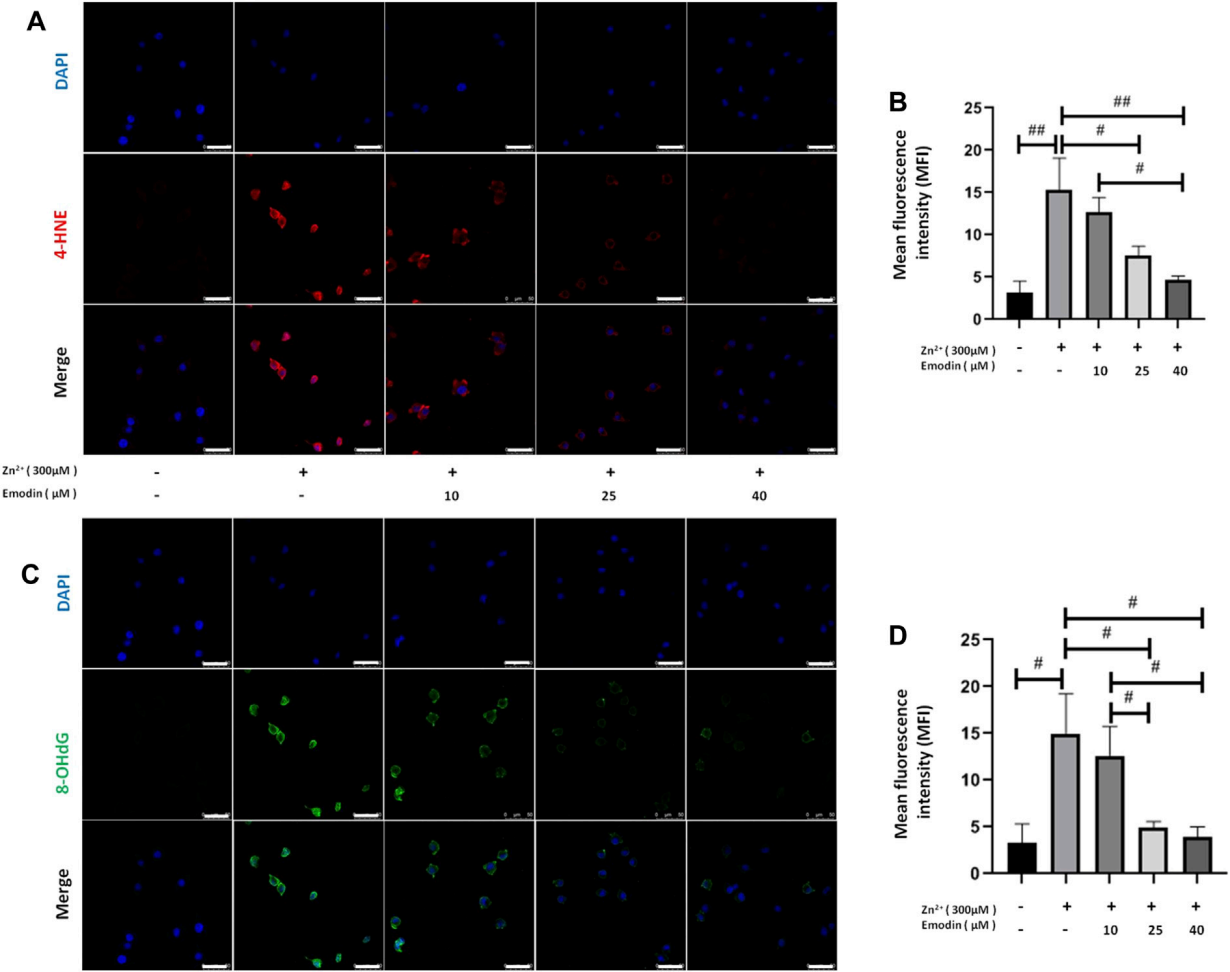
Emodin repressed lipid peroxidation and DNA oxidation in zinc-treated SH-SY5Y cells. **(A, C)** Immunofluorescence-based confocal images of 4-hydroxynonenal (4-HNE, red) and 8-hydroxy-2′-deoxyguanosine (8-OHdG, green) in untreated and 300 μM zinc sulfate-treated cells, with or without pretreatment with different concentrations of emodin with or without pretreatment with emodin (10, 25, and 40 μM). Scale bar = 50 μm. Nuclei were stained with DAPI (blue). **(B, D)** Mean fluorescence intensity analysis. One-way ANOVA and Bonferroni post hoc analysis, #*p* < 0.05, ##*p* < 0.001; Data are presented as the mean ± SEM.

To measure the potential oxidative damage following zinc treatment, products of oxidative stress, such as 4-hydroxynonenal (4-HNE) from lipid peroxidation and 8-hydroxy-2′-deoxyguanosine (8-OHdG) from DNA oxidation, were analysed by immunostaining (Figure 4). The fluorescence intensity of 4-HNE and 8-OHdG staining in SH-SY5Y cells was quantified among the control, zinc-treated and emodin-pretreated zinc groups. A 300 μM high dose of zinc significantly increased the intensity of 4-HNE (*p* = 0.0002, vs. controls). Pretreatment with emodin at 25 and 40 μM (but not 10 μM) significantly reduced the zinc-induced increase in the fluorescence intensity of 4-HNE in SH-SY5Y cells by approximately 50% (*p* = 0.006, vs. zinc-treated control group) and 70% (*p* = 0.0006, vs. zinc-treated control group), respectively (Figures 4A,B). The effect of emodin treatment at 40 μM (but not 25 μM) was significantly higher than that at 10 μM (*p* = 0.005).

A high dose of zinc significantly increased the intensity of 8-OHdG (*p* = 0.002, compared to the controls). Pretreatment with emodin at 25 and 40 μM (but not 10 μM) significantly reduced the zinc-induced increase in the fluorescence intensity of 8-OHdG in SH-SY5Y cells by approximately 68% (*p* = 0.005, vs. zinc-treated control group) and 74% (*p* = 0.002, vs. zinc-treated control group), respectively (Figures 4C,D). The effects of emodin treatment at 25 and 40 μM were significantly greater than those at 10 μM (*p* = 0.03 and *p* = 0.01, respectively).

## Discussion

Imbalances in cerebral metal homeostasis, including Zn^2+,^ are implicated in patients with AD as well as animal models recapitulating amyloidosis pathologies (Mocchegiani et al., 2005; Wang et al., 2020). Large numbers of studies strongly imply that zinc is essential for normal neural functions, while excessive zinc is harmful to neural cells. Excessive zinc induces tau formation and aggregation of neurofibrillary tangles. However, the involvement of zinc in these potential defects and its general role in neurofibrillary tangles degeneration have not been studied. Our results revealed that treatment with a pathological dose (300 μM) of zinc increased cell death, p-ERK1/2 expression, synaptic impairment, mitochondrial dysfunction and oxidative stress in SH-SY5Y cells. Pretreatment with emodin protected against these changes induced by 300 μM zinc in SH-SY5Y cells.

Zinc plays a key role in the developing human brain and physiology and in the functions of the central nervous system (CNS). Zinc dyshomeostasis and dysfunctions has been a therapeutic target for AD and other neurodegenerative diseases (Sensi et al., 2018; Xie et al., 2020). Three pools of zinc exist in the brain: protein-bound zinc, vesicular zinc, and free zinc. Large numbers of glutamatergic neurons contain zinc (Lee et al., 2000; Tóth, 2011). Earlier studies have shown that zinc can permeate through the neuronal membrane via N-methyl-D-aspartate (NMDA) receptor-gated channels. Zinc binds to NMDA receptors, and activation of NMDA receptors facilitates Zn^2+^ influx into neurons (Wang et al., 2020). In the central nervous system, 80–90% of zinc binds tightly to proteins or enzymes to maintain enzyme activity and structural stability, and the remaining 10–20% of free zinc is mainly stored in the synaptic vesicles of glutamatergic neurons (>100 μM) and is released by synapses during neuronal activity (Paoletti et al., 2009). The concentration of zinc is approximately 150 μM in the brain from healthy controls, whereas the zinc concentrations in Aβ plaques from the brains of AD patients are increased by 3-fold compared with controls (reaching more than 400 μM) (Lovell et al., 1998). Thus, in the present study, we used 300 μM zinc to mimic the concentration under pathological conditions, which was characterized in detail in a previous study (Tang et al., 2013).

Accumulating evidence has indicated that excessive zinc can stimulate the hyperphosphorylation of tau to an AD-like state by activating ERK1/2 (An et al., 2005). Zinc is also involved in several important signaling pathways, inhibits protein phosphatase 2A (PP2A) (Sun et al., 2012) and activates glycogen synthase kinase-3β (GSK-3β), ERK1/2 and c-Jun N-terminal kinase (JNK) (An et al., 2005; Kim et al., 2011; Huang et al., 2014). Here, we found that zinc treatment increased the expression of p-ERK but not total ERK, which was entirely abolished by pretreatment with emodin. This implied that emodin may have potential as a therapeutic drug for decreasing high zinc level-induced neurotoxicity by suppressing p-ERK1/2.

Zinc is involved in the regulation of synaptic functions (Yin et al., 2002). Zn^2+^ released from synapses induces tau hyperphosphorylation through many signaling pathways, including the Src-dependent tyrosine kinase family and the GSK-3β and ERK pathways (Xiong et al., 2013). *In vivo* studies in animal models have shown that the chelator-driven perturbation of Zn^2+^ in the brain decreased the levels of brain-derived neurotrophic factor, PSD95, and dendritic spine density (Frazzini et al., 2018; Lai et al., 2021). Reduced levels of synaptic-related proteins indicated decreased synaptic function in response to zinc exposure. Our results indicated a protective effect exerted by emodin on synaptic impairment, in line with an earlier study (Lai et al., 2020). However, the optimal dosage of emodin against pre- and postsynaptic proteins differs in our observations. The highest dose of emodin (40 μM) significantly alleviated the zinc-induced reduction in presynaptic proteins but did not ameliorate the reduction in postsynaptic PSD95.

In addition to affecting synaptic function, zinc has been shown to be linked with oxidative stress and neuroinflammation, which are important in AD disease development (Tönnies and Trushina, 2017; Ni et al., 2019; Rivers-Auty et al., 2021). In the present study, pretreatment of SH-SY5Y cells with emodin suppressed zinc-induced ROS generation, reduced the formation of a lipid peroxidation product (4-HNE), and decreased the level of a DNA damage marker (8-OHdG). These results suggest that emodin has an antioxidant effect on zinc-induced ROS generation. Free radicals are harmful components causing lipid peroxidation, DNA damage, and protein oxidation in neurons (Giordano et al., 2014). ROS is an important trigger of neuronal apoptosis. The decrease in mitochondrial ΔΨm is a sign of early apoptosis. We found that pretreatment with emodin reestablished the mitochondrial membrane potential, which contributed to suppressing the ERK1/2 signaling pathway. In the present study, zinc-induced ROS generation resulted in a dissipation of ΔΨm, indicating mitochondrial dysfunction. Zinc-induced neurotoxicity has been reported to be associated with ROS generation (Wang et al., 2020). The mitochondrial respiratory chain, which regulates apoptosis, is susceptible to Zn^2+^. The accumulation of Zn^2+^ further induces mitochondrial dysfunction and oxidative stress (Furuta et al., 2016). Free Zn^2+^ induces mitochondrial permeability transition and the generation of ROS (Bossy-Wetzel et al., 2004). ROS can, in turn, increase the detrimental amount of zinc released from metallothioneins (Aizenman et al., 2010), forming a vicious cycle.

Earlier studies have shown a role of emodin in reducing Aβ-induced cytotoxicity and deposition and tau hyperphosphorylation (Liu et al., 2010b; Sun and Liu, 2015; Du et al., 2019; Zeng et al., 2019; Wang and Liu, 2020; Li et al., 2021) and offering neuroprotective, anti-inflammatory and antioxidant effects (Park et al., 2016). Emodin may additionally act through the PI3K pathway, Akt/GSK-3β pathways and estrogen receptor ErbB1/ErbB2 (Mizuno et al., 2008; Liu et al., 2010b; Sun and Liu, 2015; Yi et al., 2016). Here, we investigated only the involvement of the ERK-1/2 signaling pathway (Leung et al., 2020). A previous study by our group showed the neuroprotective effect of emodin via the ERK1/2 signaling pathway and alleviated oxidative stress and mitochondrial dysfunction. We do not know if/how this effect is effective on oxidative stress in AD. Liu et al. previously reported that emodin inhibited the influx of Zn^2+^ (200 μM) into neuronal cells, thereby preventing the consumption of nicotinamide adenine dinucleotide and adenosine triphosphate, inhibiting the generation of ROS and endoplasmic reticulum stress, and inactivating AMP-activated protein kinase (AMPK)/acetyl-CoA carboxylase (ACC) signaling pathways to exert neuroprotective effects (Zhao et al., 2019). In the current study, our data show that emodin has neuroprotective effects, while 300 μM zinc reaches a pathological concentration of AD, which has been characterized previously (An et al., 2005; Tang et al., 2013). Liu et al. used a 200 μM Zn^2+^ concentration from GT1-7 cells (immortalized hypothalamic neurons) (Kawahara et al., 2013). Our previous data showed that the concentration of zinc (200 µM) and the treatment time (1 h) selected by Liu et al. did not significantly influence the viability of SH-SY5Y cells, while 300 μM Zn^2+^ for 4 h was sufficient to decrease the viability of SH-SY5Y cells (An et al., 2005). In addition, we observed that emodin (10–40 μM) protected against zinc-induced cell death in SH-SY5Y cells. Zinc causes tau phosphorylation in a time- and dose-dependent manner (An et al., 2005; Tang et al., 2013), while oxidative stress and synaptic damage are related to tau phosphorylation. Finally, our data showed the neuroprotective effect of emodin against pathological concentrations of zinc by inhibiting oxidative stress, which is achieved through the ERK1/2 pathway. Further studies are needed to elucidate the signaling pathway that may mediate the effect of emodin in the central nervous system in a more systematic manner.

## Conclusion

In conclusion, we demonstrated a protective effect of emodin against zinc-induced neurotoxicity in the SH-SY5Y cell line. Emodin pretreatment normalized zinc-induced synaptic impairment, reduced oxidative stress through the inhibition of the phospho-ERK1/2 signaling pathway, and inhibited mitochondrial dissipation. Further studies are needed to investigate the neuroprotective effects of emodin and the pathways involved in animal models with pathological accumulation of zinc in the brain.

## Data Availability

The original contributions presented in the study are included in the article/Supplementary Material, further inquiries can be directed to the corresponding authors.
