# Supplementary figures and images for: Emodin Protects SH-SY5Y Cells Against Zinc-Induced Synaptic Impairment and Oxidative Stress Through the ERK1/2 Pathway
Source: Front Pharmacol. 2022 Feb 7;13:821521. doi: 10.3389/fphar.2022.821521 (PMC8859424; doi:10.3389/fphar.2022.821521)

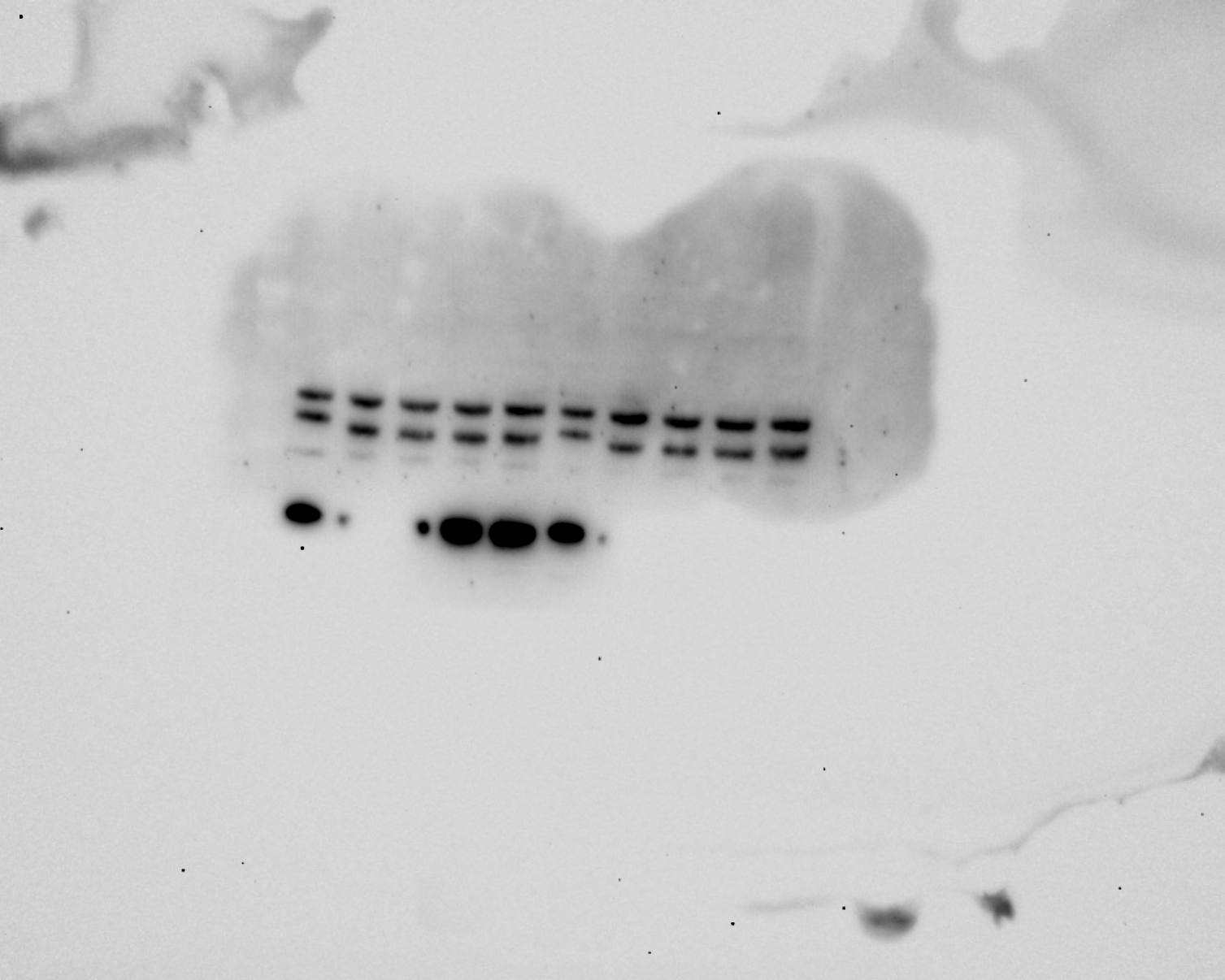

Supplement: Supplementary file 1 [file DataSheet1.ZIP › original data of gel and membrane/p-Erk-(Chemiluminescence)-1.tif]

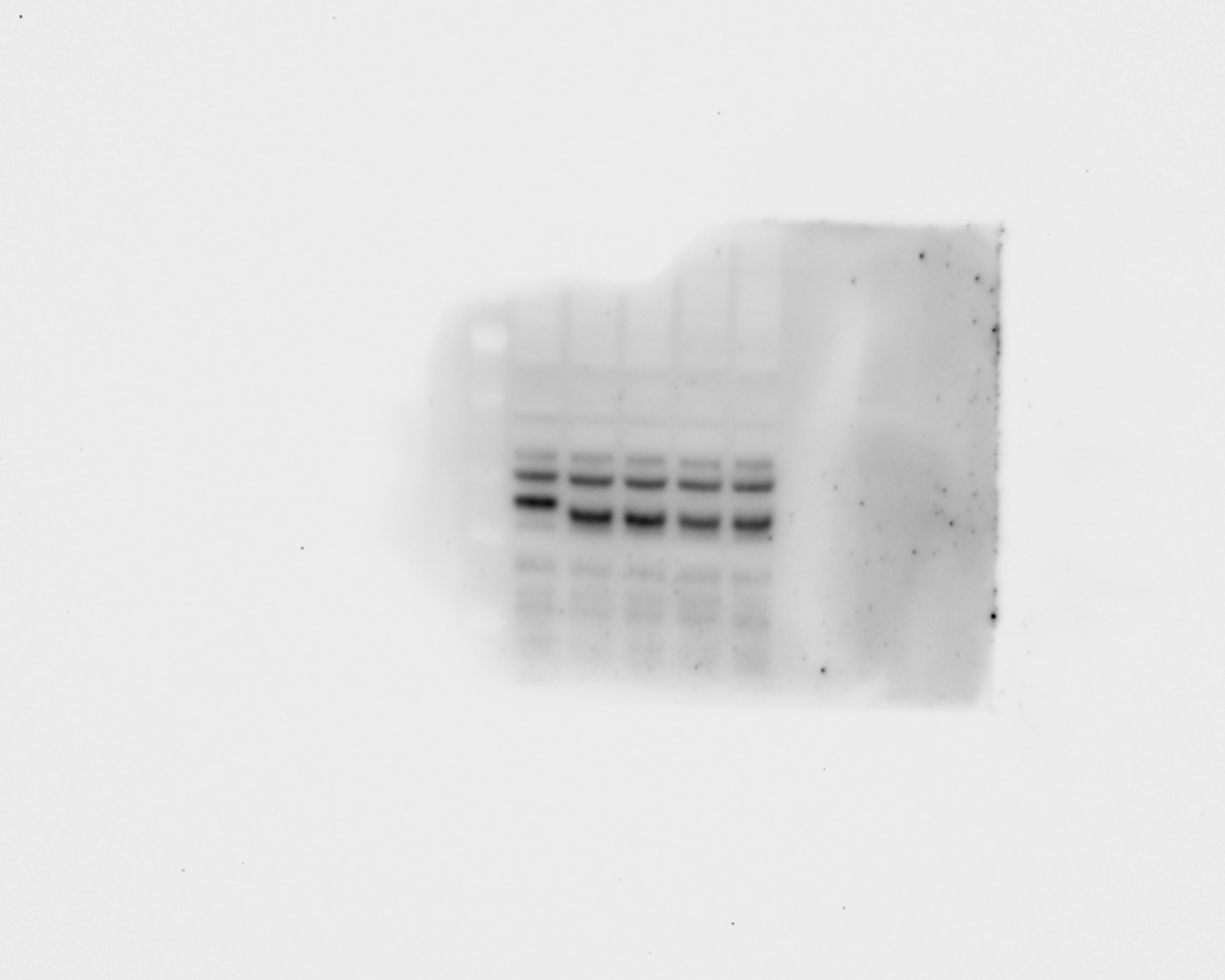

Supplement: Supplementary file 1 [file DataSheet1.ZIP › original data of gel and membrane/p-Erk-(Chemiluminescence)-2.tif]

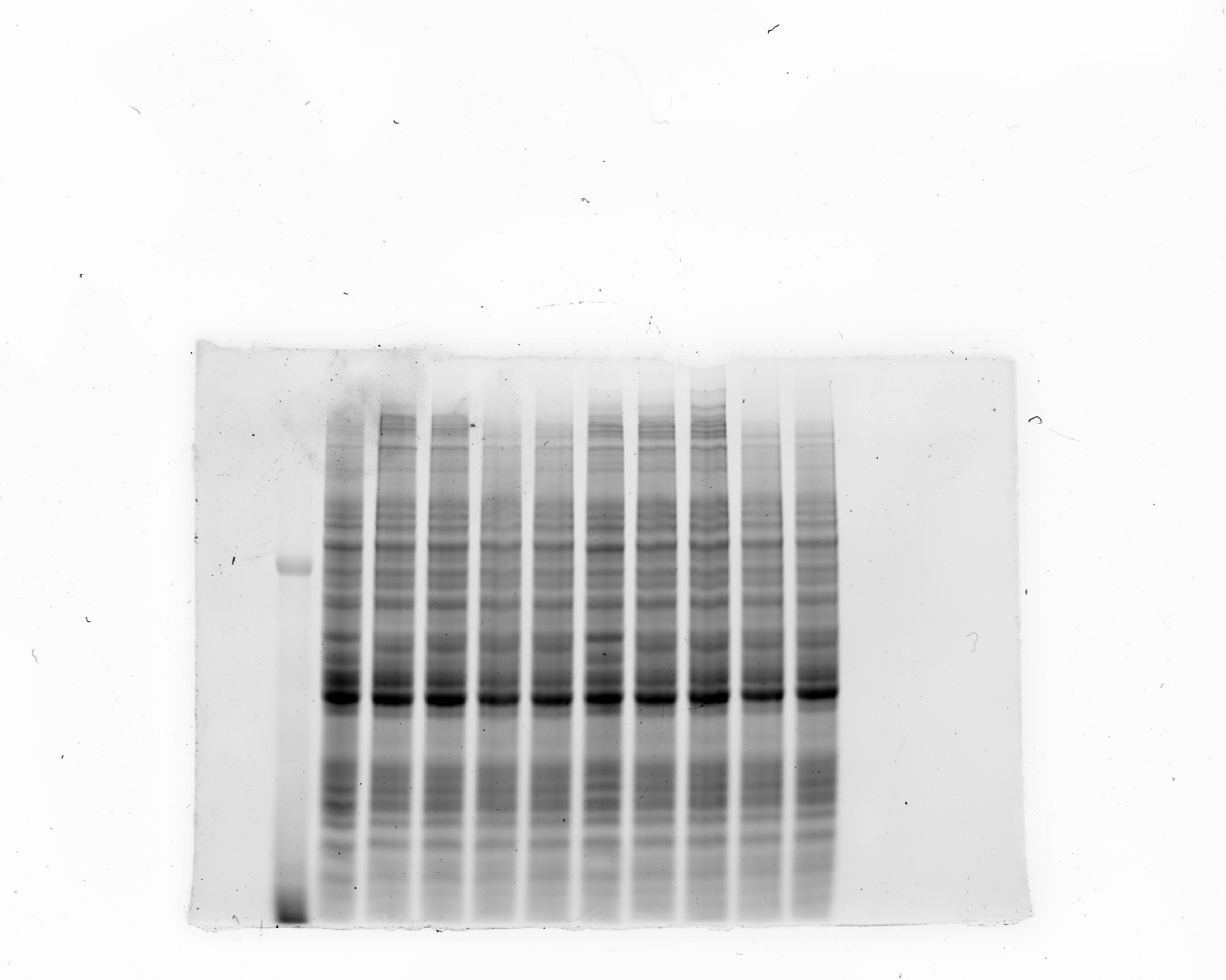

Supplement: Supplementary file 1 [file DataSheet1.ZIP › original data of gel and membrane/p-ERK-(Stain Free Gel)-1.tif]

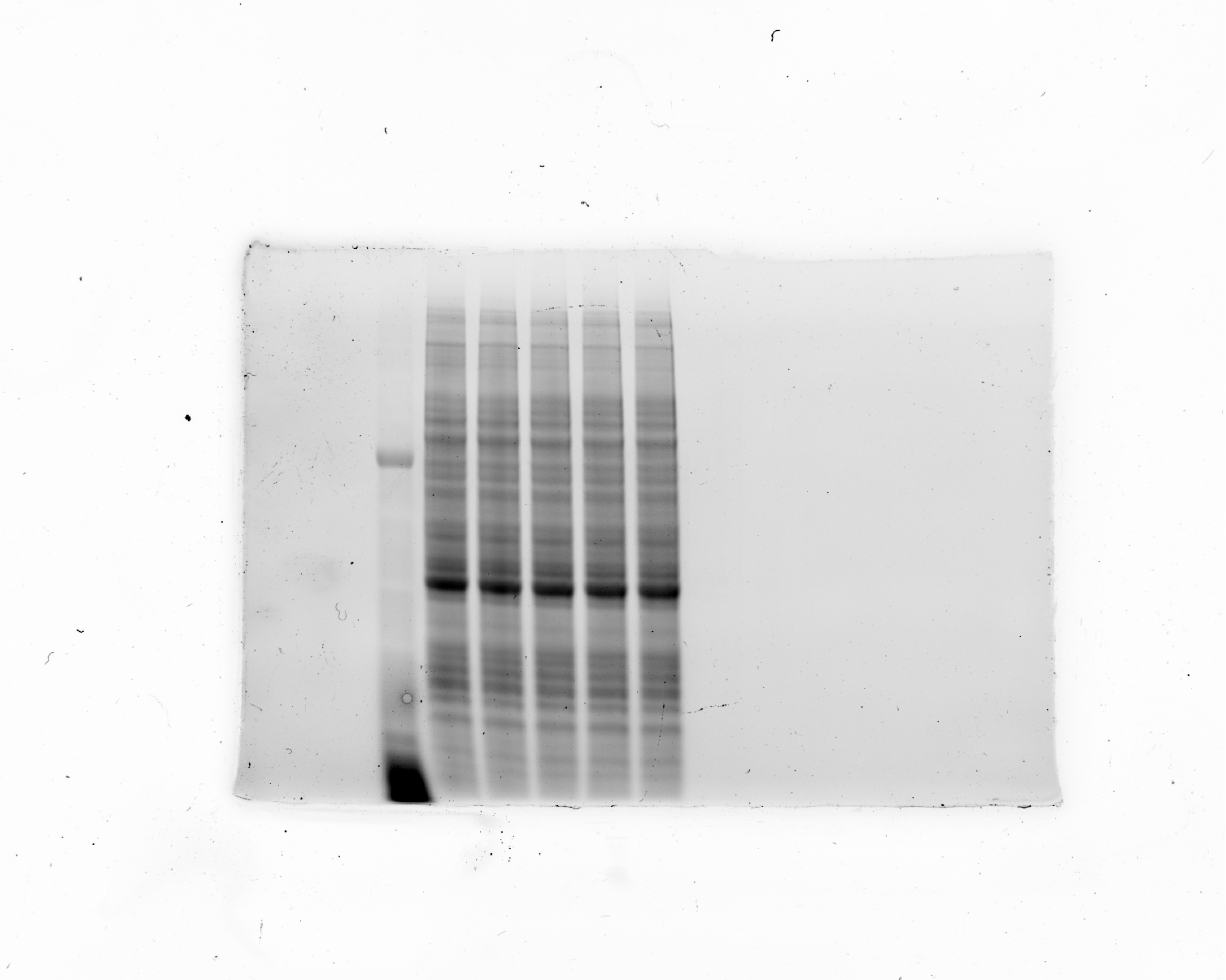

Supplement: Supplementary file 1 [file DataSheet1.ZIP › original data of gel and membrane/p-ERK-(Stain Free Gel)-2.tif]

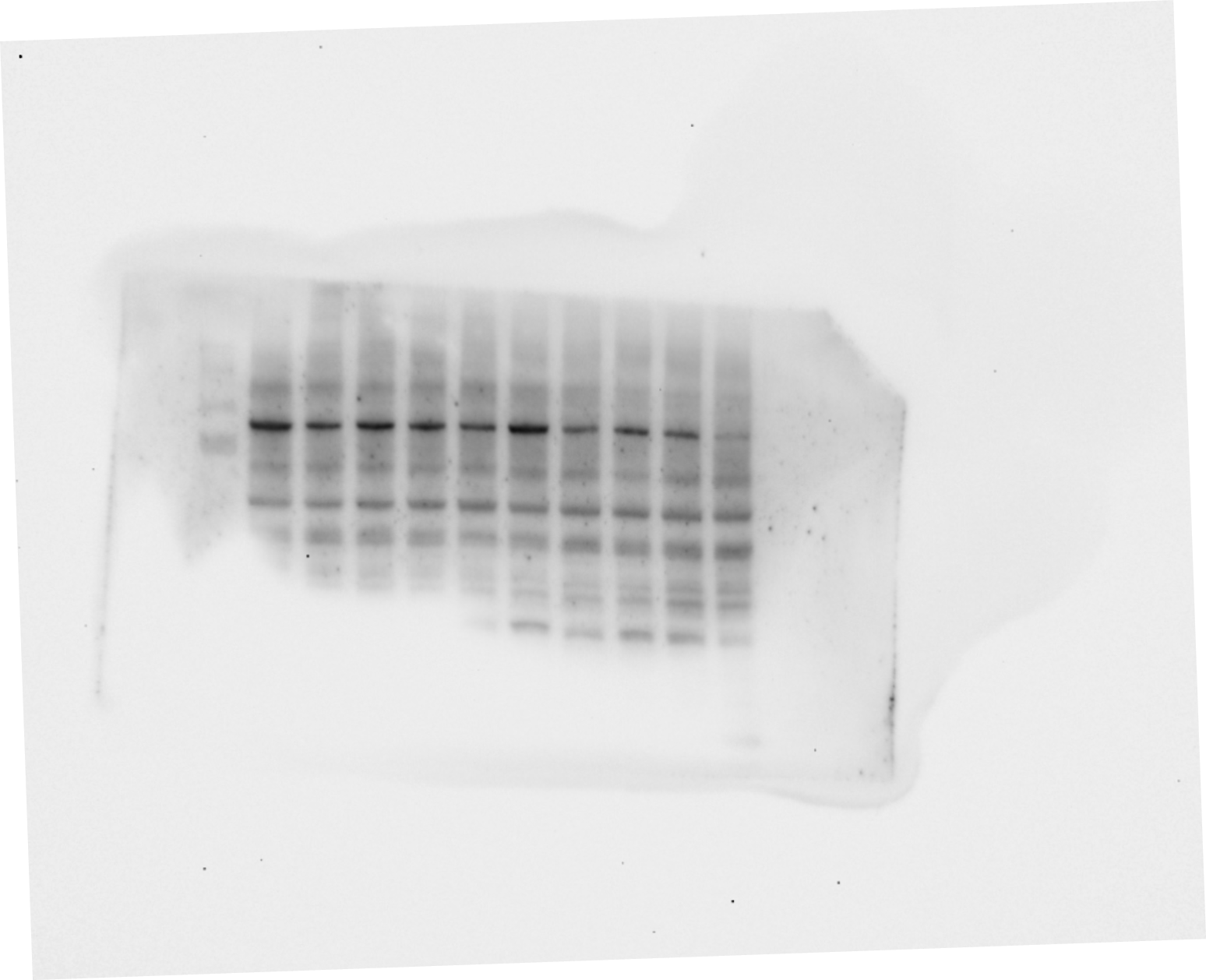

Supplement: Supplementary file 1 [file DataSheet1.ZIP › original data of gel and membrane/PSD95(Chemiluminescence)-1.tif]

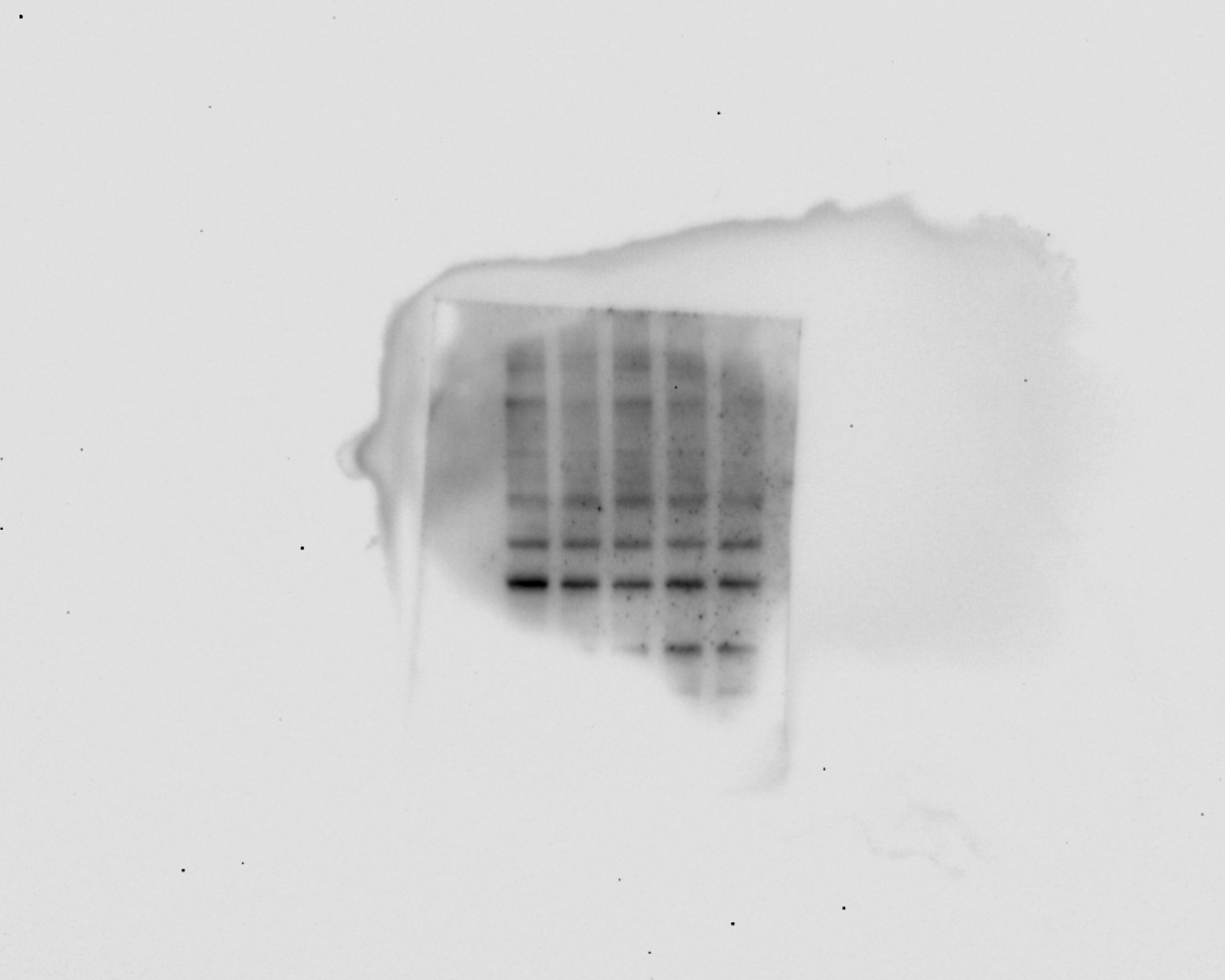

Supplement: Supplementary file 1 [file DataSheet1.ZIP › original data of gel and membrane/PSD95(Chemiluminescence)-2.tif]

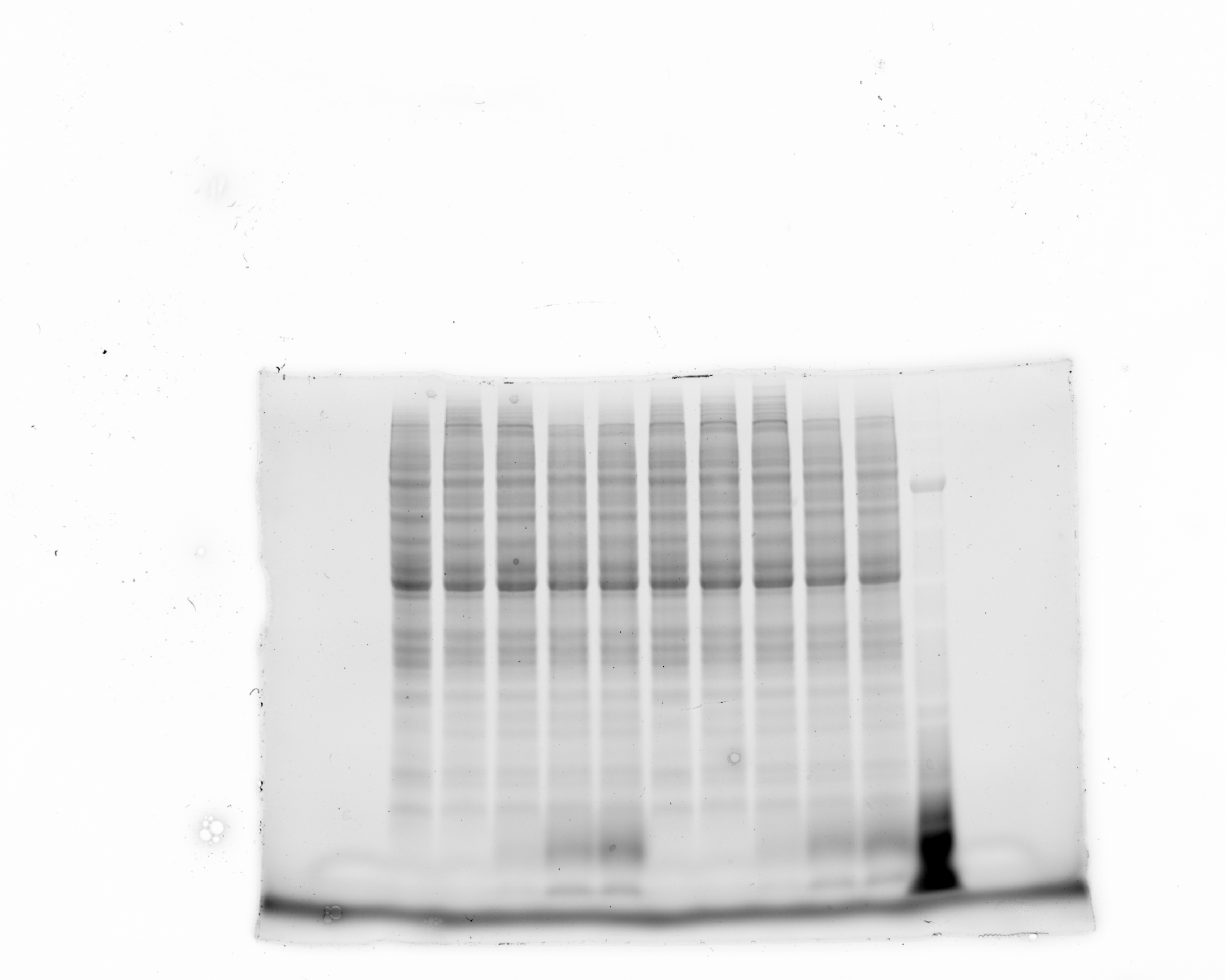

Supplement: Supplementary file 1 [file DataSheet1.ZIP › original data of gel and membrane/PSD95-(Stain Free Gel)-1.tif]

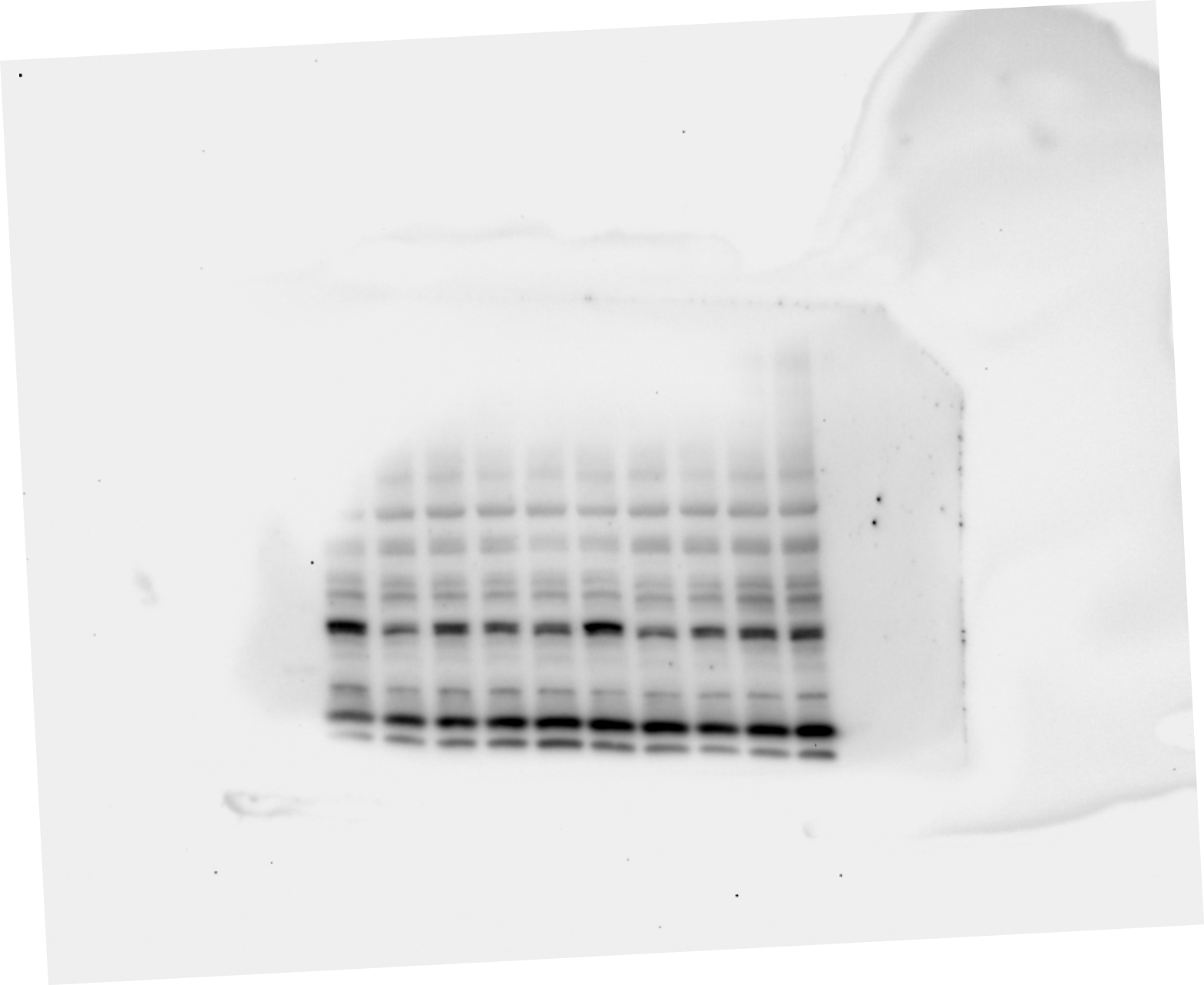

Supplement: Supplementary file 1 [file DataSheet1.ZIP › original data of gel and membrane/SNAP25(Chemiluminescence)-1.tif]

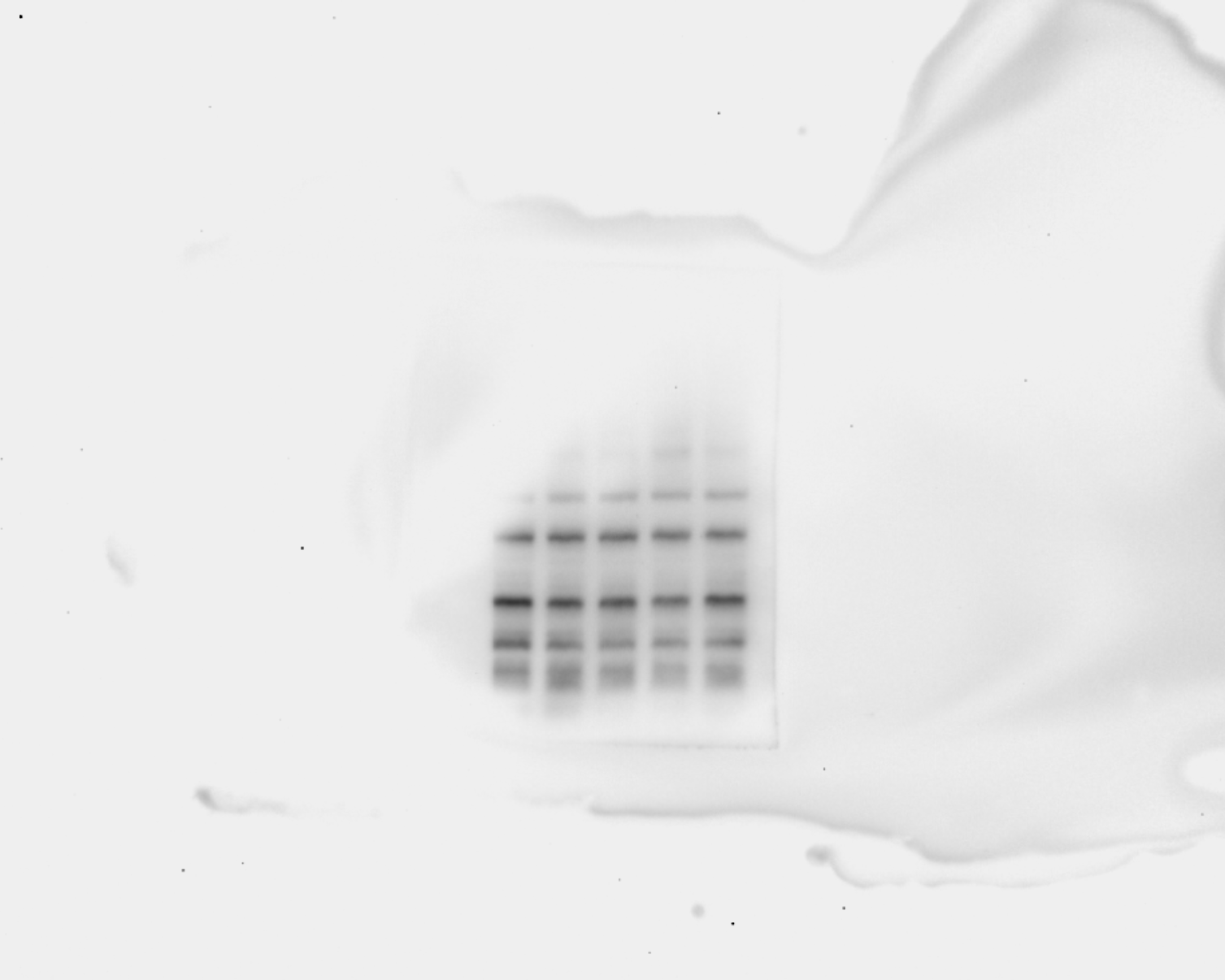

Supplement: Supplementary file 1 [file DataSheet1.ZIP › original data of gel and membrane/SNAP25(Chemiluminescence)-2.tif]

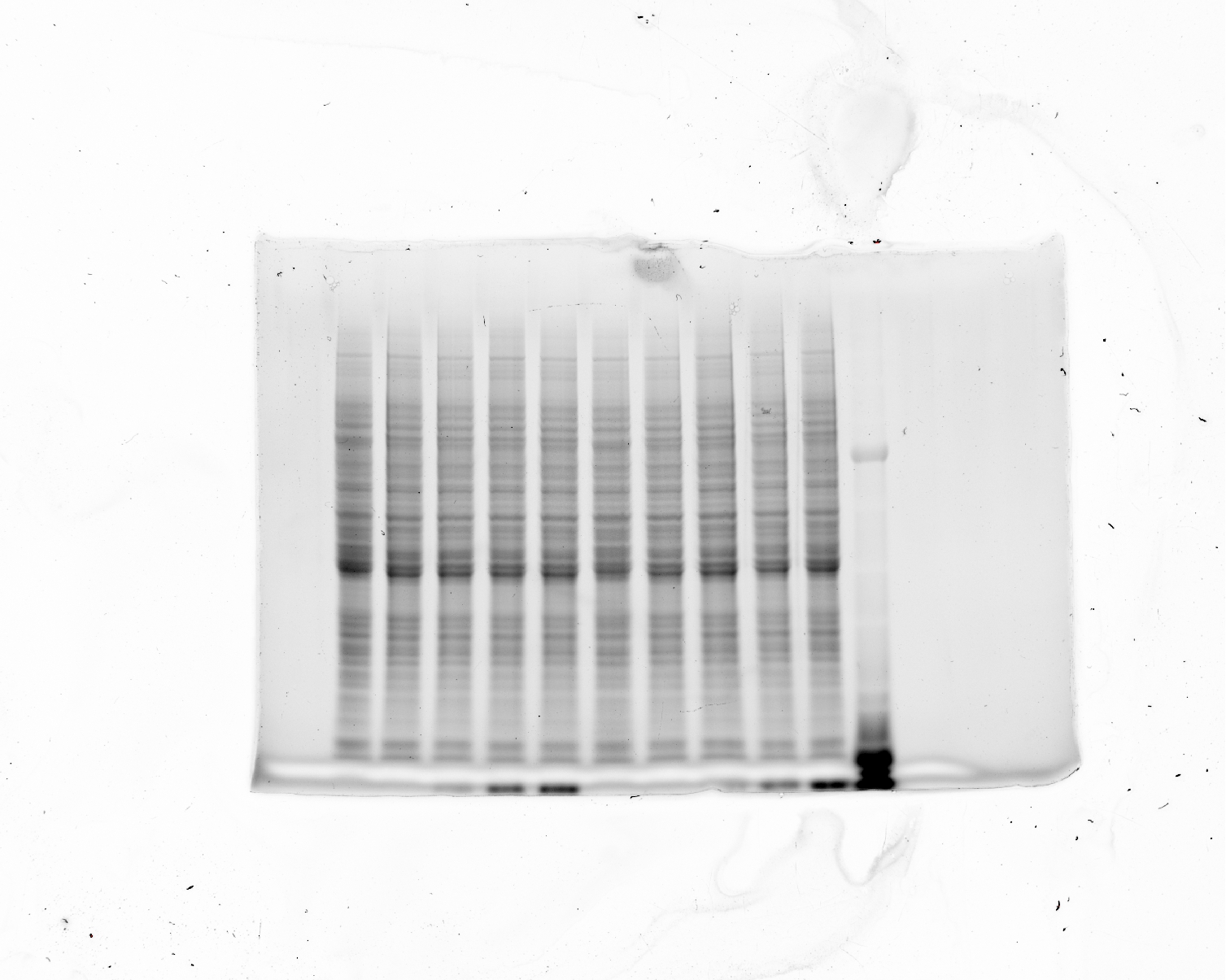

Supplement: Supplementary file 1 [file DataSheet1.ZIP › original data of gel and membrane/SNAP25-(Stain Free Gel)-1.jpg]

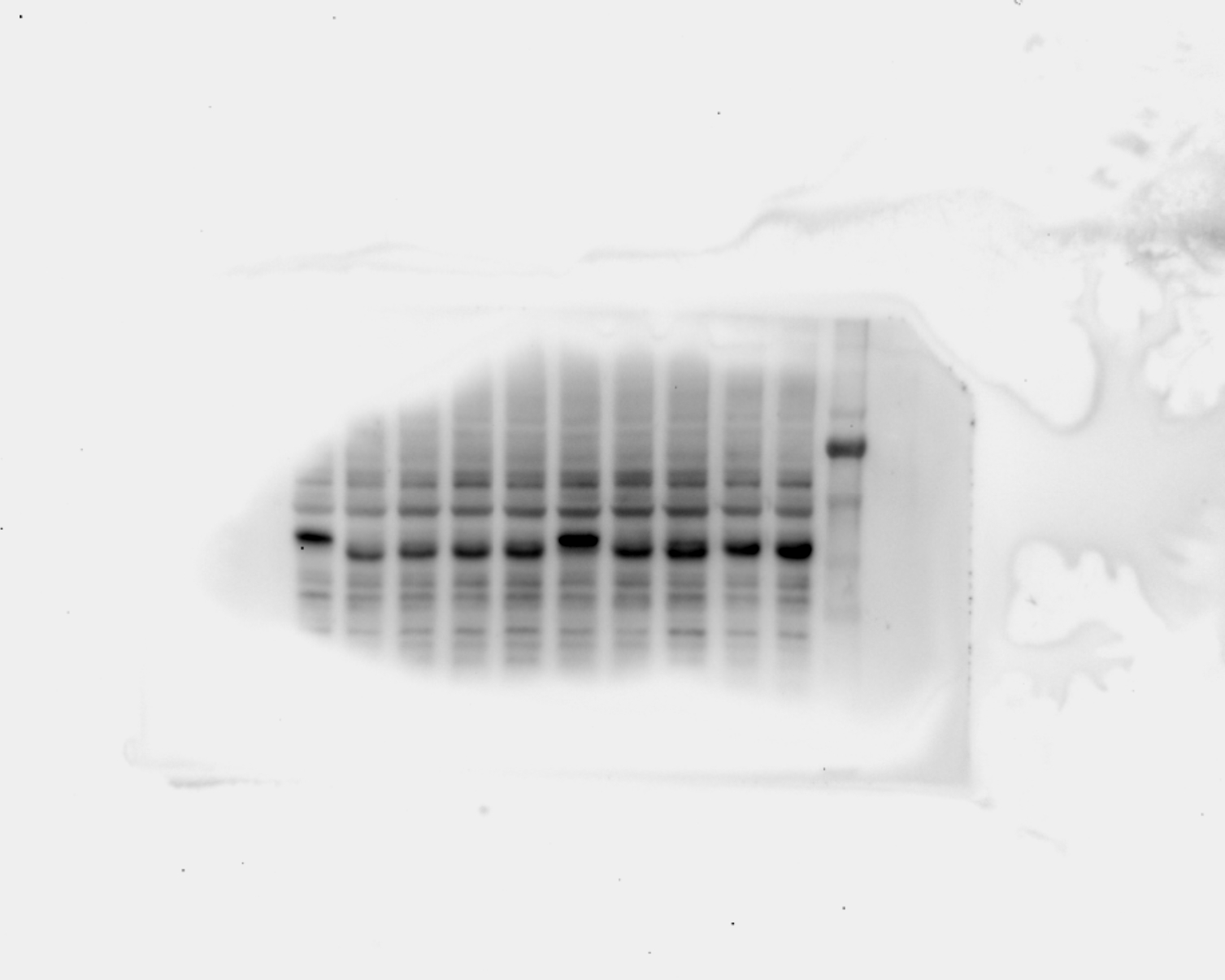

Supplement: Supplementary file 1 [file DataSheet1.ZIP › original data of gel and membrane/synaptophysin(Chemiluminescence)-1.jpg]

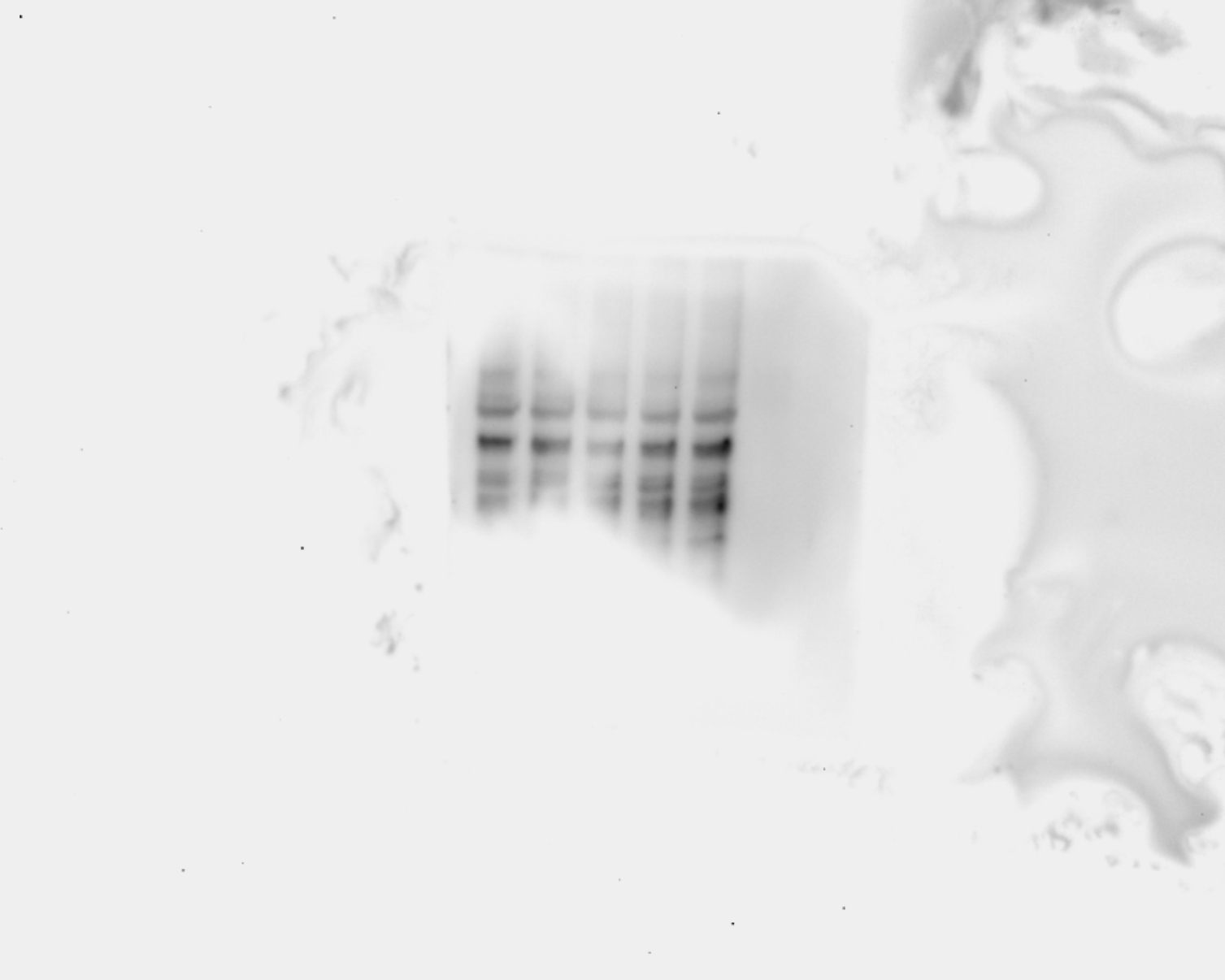

Supplement: Supplementary file 1 [file DataSheet1.ZIP › original data of gel and membrane/synaptophysin(Chemiluminescence)-2.tif]

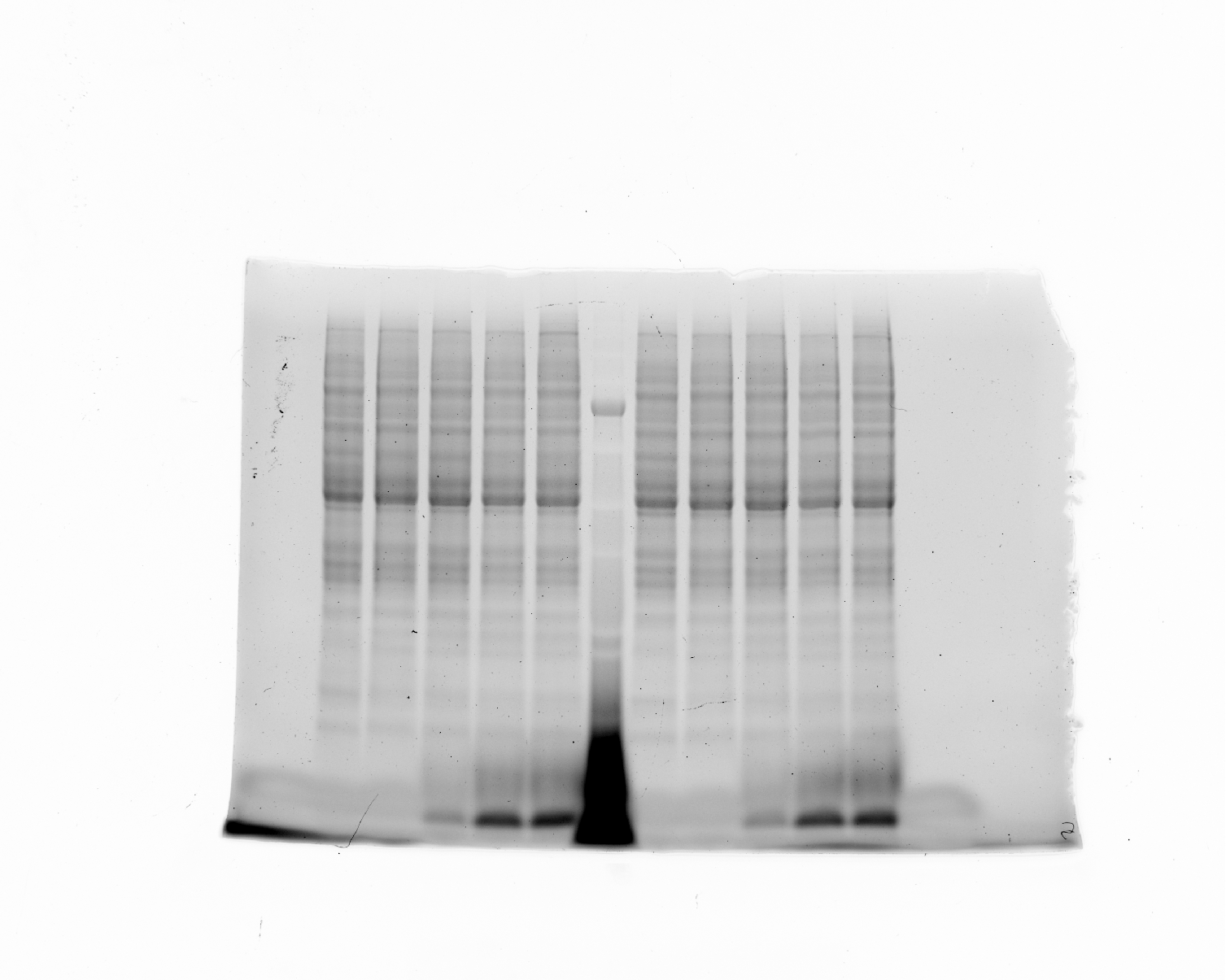

Supplement: Supplementary file 1 [file DataSheet1.ZIP › original data of gel and membrane/synaptophysin(right)-PSD95(left)-(Stain Free Gel)-2.tif]

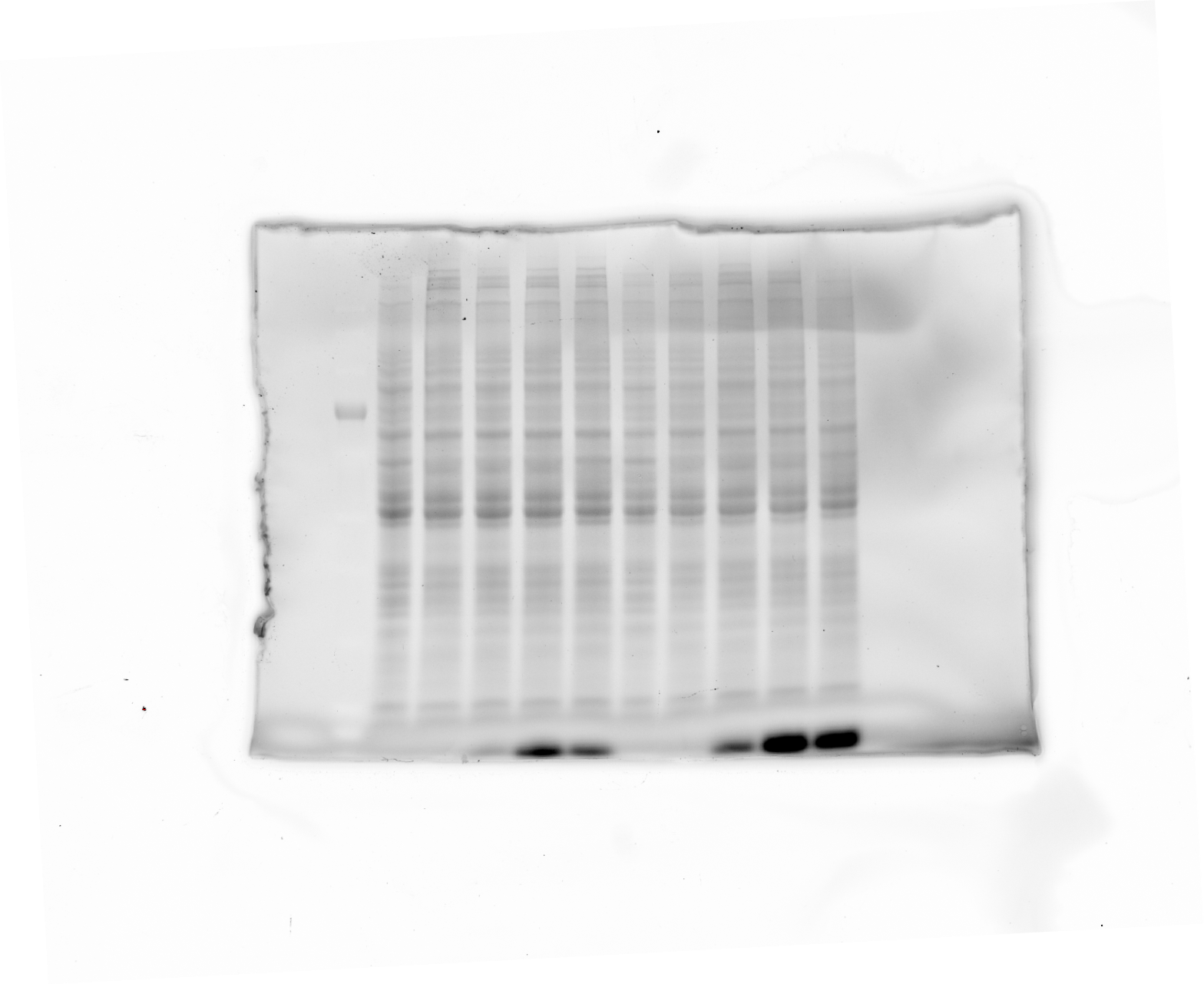

Supplement: Supplementary file 1 [file DataSheet1.ZIP › original data of gel and membrane/synaptophysin-(Stain Free Gel)-1.tif]

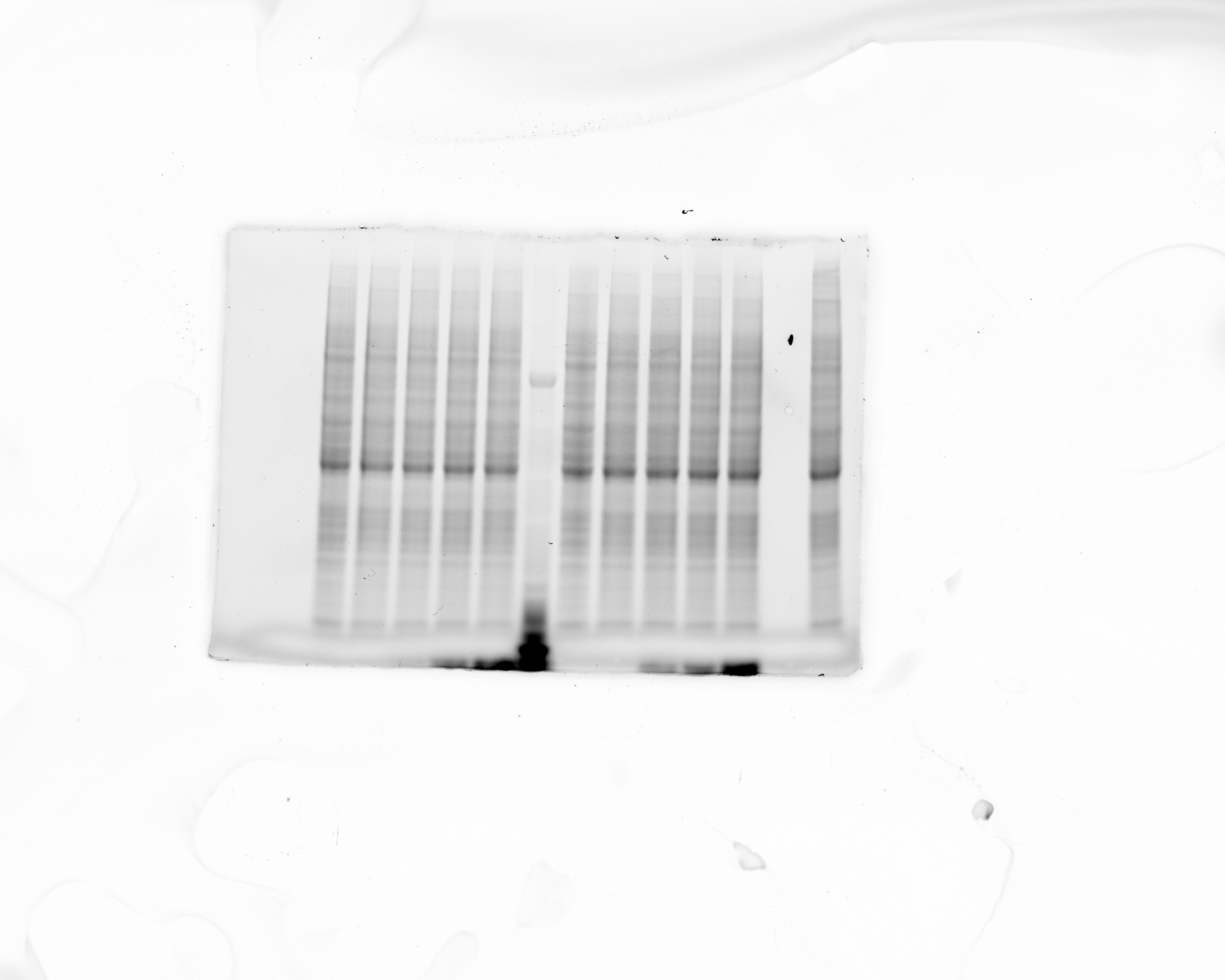

Supplement: Supplementary file 1 [file DataSheet1.ZIP › original data of gel and membrane/T-ERK(left)-SNAP25(right)-(Stain Free Gel)-2.jpg]

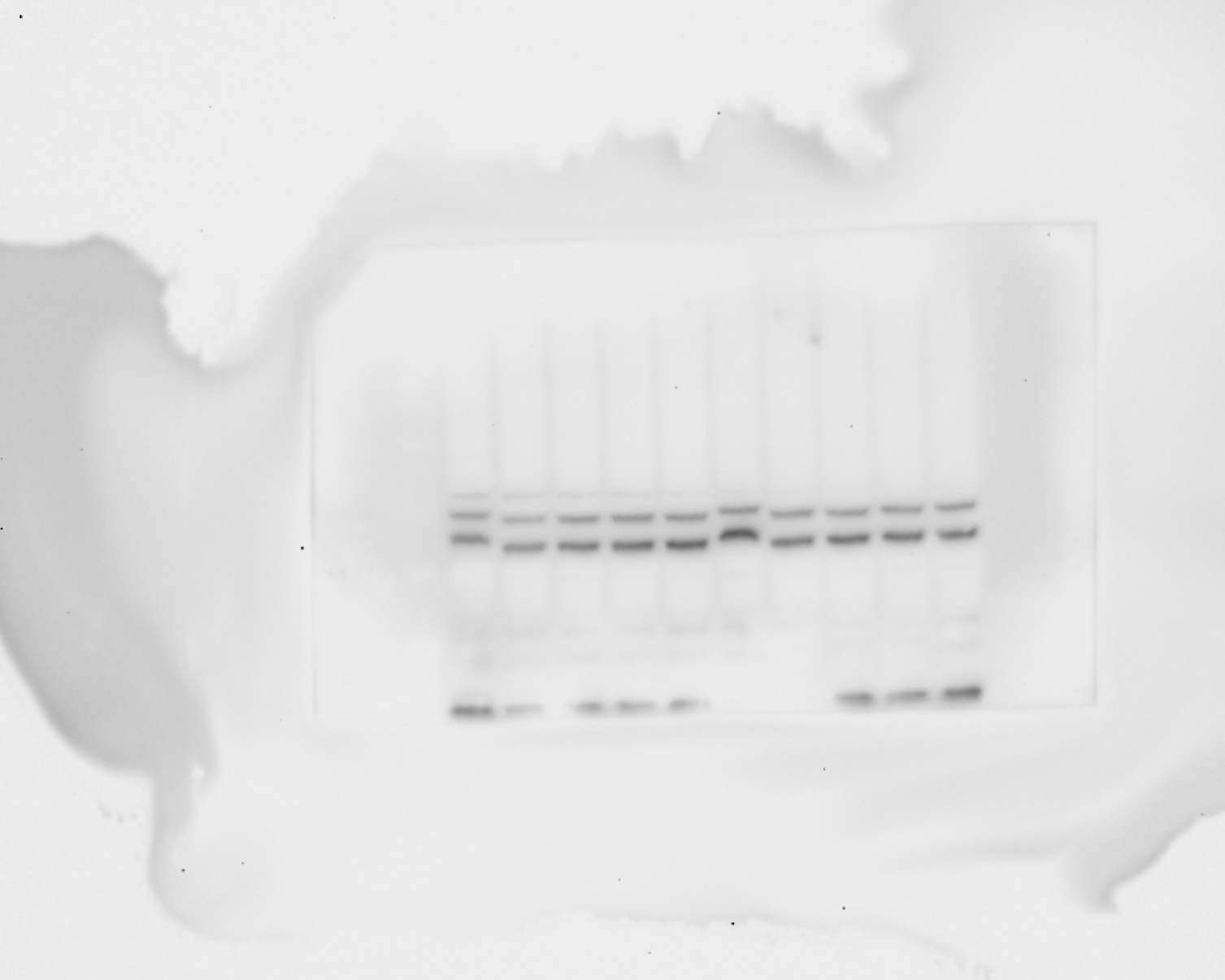

Supplement: Supplementary file 1 [file DataSheet1.ZIP › original data of gel and membrane/T-Erk-(Chemiluminescence)-1.tif]

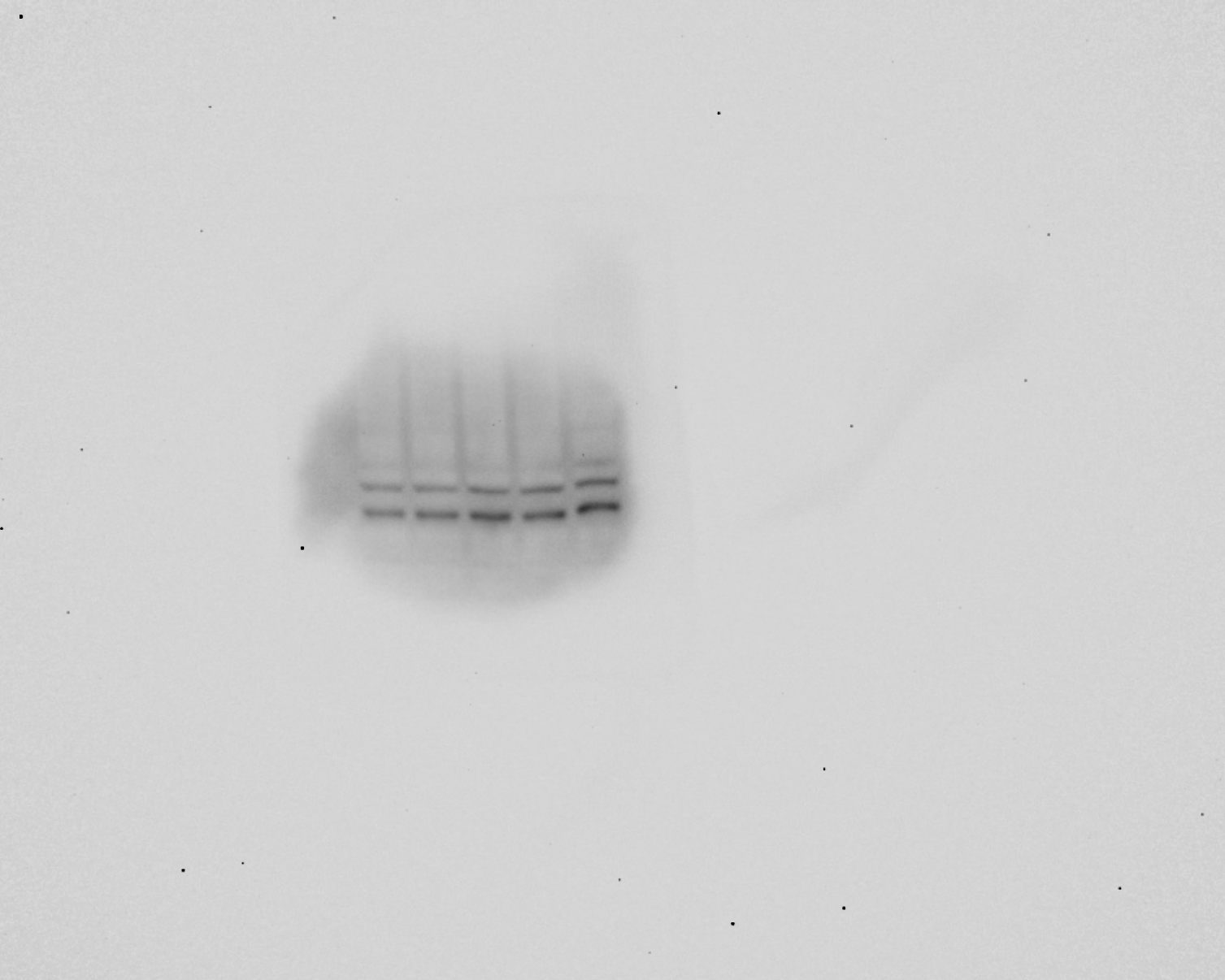

Supplement: Supplementary file 1 [file DataSheet1.ZIP › original data of gel and membrane/T-Erk-(Chemiluminescence)-2.tif]

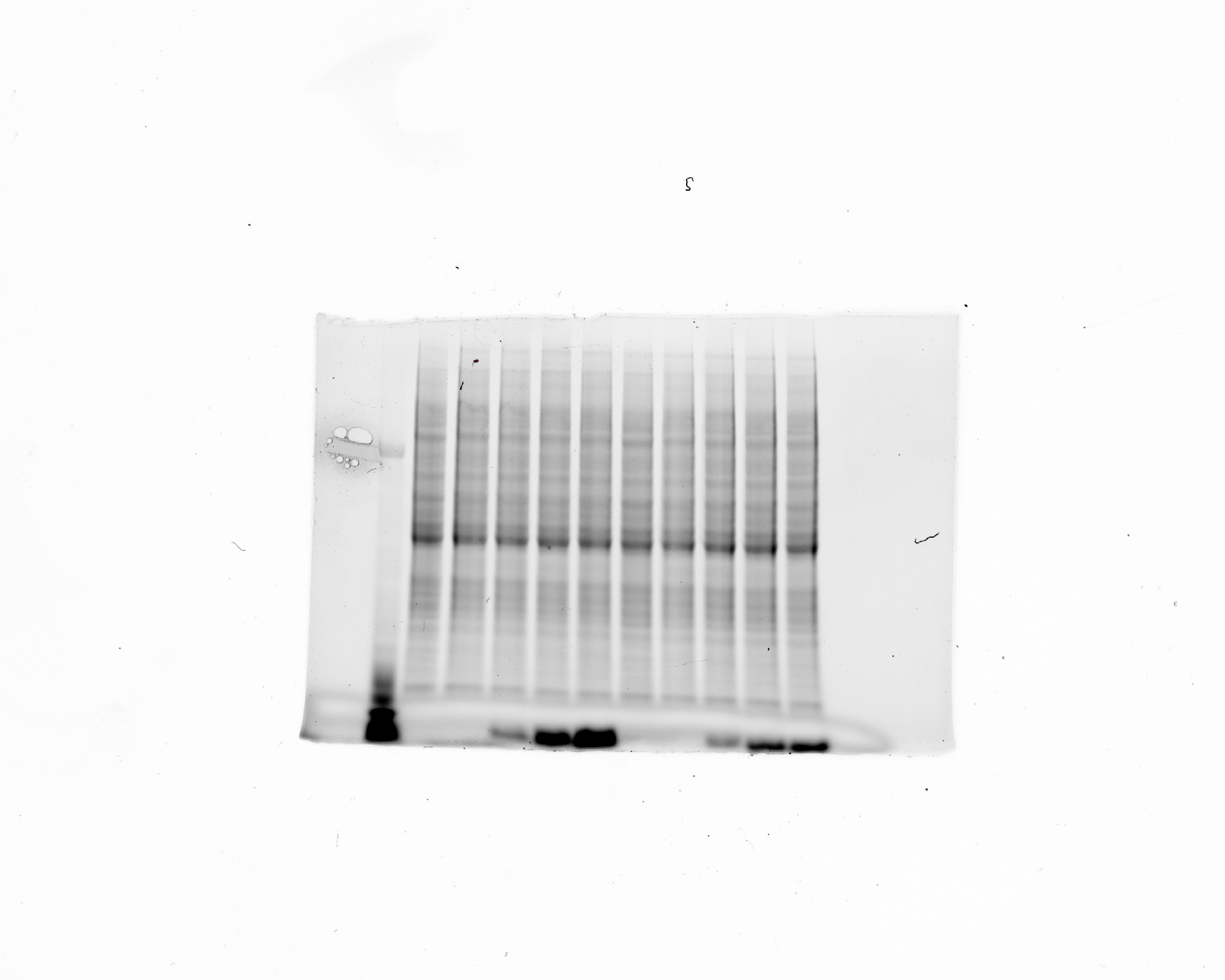

Supplement: Supplementary file 1 [file DataSheet1.ZIP › original data of gel and membrane/T-ERK-(Stain Free Gel)-1.jpg]
